# Fractal-Based Thermal Conductivity Prediction Modeling for Closed Mesoporous Polymer Gels

**DOI:** 10.3390/gels11060391

**Published:** 2025-05-26

**Authors:** Haiyan Yu, Mingdong Li, Ning Guo, Anqi Chen, Haochun Zhang, Mu Du

**Affiliations:** 1 Institute of Thermal Science and Technology, Shandong University, Jinan 250061, China; 202414341.sdu@vip.163.com (M.L.); 202334300@mail.sdu.edu.cn (N.G.); 202234272@mail.sdu.edu.cn (A.C.); 2 School of Energy Science and Engineering, Harbin Institute of Technology, Harbin 150001, China; hczhang@hit.edu.cn; 3 Institute for Advanced Technology, Shandong University, Jinan 250061, China

**Keywords:** thermal conductivity, microscale thermal radiation, mesoporous polymer gels, fractal modeling, microscale heat transfer

## Abstract

The closed mesoporous polymer gels have garnered significant attention as advanced thermal insulation materials due to their superior lightweight characteristics and excellent thermal management capabilities. To accurately predict their thermal performance, this study develops a novel mathematical model that integrates fractal geometry theory, Kirchhoff’s thermal conduction principles, comprehensive Rosseland diffusion approximation, and Mie scattering theory. The conductive thermal conductivity component was formulated based on a diagonal cross fractal structure, while the radiative component was derived considering microscale radiative effects. Model predictions exhibit strong agreement with experimental results from various mesoporous polymer gels, achieving a prediction error of less than 11.2%. Furthermore, a detailed parametric analysis was conducted, elucidating the influences of porosity, cell size, temperature, refractive index, and extinction coefficient. The findings identify a critical cell size range (1–100 µm) and porosity range (0.74–0.97) where minimum thermal conductivity occurs. This proposed modeling approach offers a robust and efficient theoretical tool for designing and optimizing the thermal insulation characteristics of closed mesoporous polymer gels, thereby advancing their application in diverse energy conversion and management systems.

## 1. Introduction

The development of high-performance thermal insulation materials is paramount for addressing global energy efficiency challenges. Among emerging materials, polymer gels with closed mesoporous structures have garnered significant interest [1,2], leading to their widespread application in energy management and conversion devices [3,4,5]. To further expand the application of these materials, accurate and efficient predictive methods for their thermal conductivity are crucial. Heat transfer within closed mesoporous polymer (CMP-) gels primarily occurs through thermal conduction via the solid polymer matrix and the entrapped gas within the pores, and thermal radiation across the porous structure. Due to the confined nature of the mesopores (typically < 50 nm) and small cell sizes, convective heat transfer is generally considered negligible [6,7]. The existing research on the conductive equivalent thermal conductivity (*κ*_cond_) of porous polymer gels is mainly divided into three categories: (i) Empirical models [8,9], which depend significantly on experimental data and vary with material properties and cell structures. (ii) Equivalent circuit methods, where the conductive effective thermal conductivity is derived analytically by modeling thermal resistance networks within periodic cell structures composed of struts and walls [10]. By taking the phonon scattering effect [11,12] of skeleton solid phases and the Knudsen effect [13] of pore gaseous phases into account, an improved equivalent circuit method for closed mesoporous polymer gels has been proposed for the thermal conductivity [11,14]. (iii) Equivalent fractal method [15,16], based on fractal geometry theory, are utilized for modeling thermal conductivity in disordered, scale-invariant structures [17,18,19]. (iv) Numerical simulation method, involving computational models generated through image-based fitting or geometric simplification. These models typically employ numerical techniques such as Finite Element Analysis (FEA) [20,21], molecular dynamics [22], Lattice Boltzmann method [23,24] to solve heat conduction problems numerically.

In contrast to conduction, fewer studies have focused on radiative equivalent thermal conductivity (*κ*_rad_) in porous dielectric materials, particularly at microscale dimensions, even though radiative heat transfer becomes increasingly significant as cell sizes decrease [12]. Existing radiative thermal conductivity models for CMP-gels can generally be categorized into four types: (i) Radiative Transfer Equation (RTE) Solvers. While accurate, solving the full RTE is computationally intensive and often limited to simplified geometries. [25,26,27]. This geometric simplification limits the scope of application of this method. (ii) Approximations Ignoring Processes. Models sometimes neglect absorption or scattering terms, limiting their applicability [25]. Obviously, the scope of application of this model is limited. (iii) Rosseland diffusion approximation [26,28]. This widely used method relates *κ*_rad_ to the material’s extinction coefficient. However, at the microscale, the thermal radiative energy significantly exceeds the value calculated by the blackbody radiation equation, rendering the Rosseland diffusion approximation inapplicable at the micro/nanoscale. (iv) Microscale radiation method. Attempts have been made to modify the Rosseland approximation by incorporating correction factors or considering near-field effects, but a unified approach remains elusive [14,25,29,30]. Consequently, developing a precise model for the radiative thermal conductivity of CMP-gels is essential.

This research provides a theoretically grounded and computationally efficient tool for predicting thermal transport in CMP-gels. The conductive equivalent thermal conductivity equation was generated based on the Diagonal Cross Fractal (DCF) model and Kirchhoff’s law, while the radiative thermal conductivity was derived from the full-scale Rosseland diffusion and the Mie theory. Then, according to this mathematical prediction method of calculating thermal conductivity, five impact factors on the thermal conductivity, including porosity, cell size, temperature, the material’s refractive index, and the material’s extinction coefficient, were extensively investigated. This mathematical prediction method offers a convenient approach for predicting both the conductive and radiative equivalent thermal conductivities of CMP-gels, which significantly improves the efficiency of thermal conductivity calculations in energy transfer and utilization equipment.

## 2. Results and Discussion

### 2.1. Model Verification

**Figure 1:**
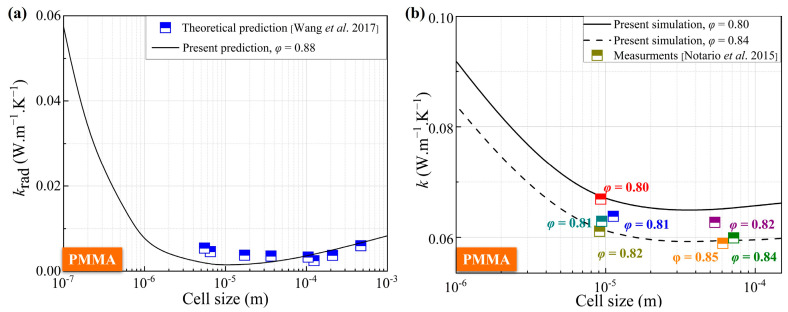
The anticipated outcomes regarding the equivalent thermal conductivity of CMP-gels in relation to cell size have been compared with empirical data: (**a**) the radiative equivalent thermal conductivity [7]; (**b**) the total equivalent thermal conductivity [13]. The points represent Notario’s experimental data, with different colors corresponding to the porosity value indicated next to each color.

The predictive capability of the DFC model was assessed by comparing its outputs against published experimental thermal conductivity data for various CMP-gels. Figure 1 illustrates these comparisons. For closed-cell polymethyl methacrylate (PMMA) gels, the model predictions show good agreement with experimental results from Wang et al. [12] and Notario et al. [31]. Specifically, predictions for *κ*_total_ at an average porosity *φ* = 0.88 match data across cell sizes from 30 μm to 450 μm with errors below 8.4%. Similarly, predictions for *φ* = 0.80 and *φ* = 0.84 align well with measurements [32], exhibiting a maximum deviation of 11.2%.

This level of agreement across different porosities and cell sizes validates the model’s ability to capture the essential physics governing heat transfer in these materials, confirming its feasibility and accuracy for predicting thermal conductivity in CMP-gels. Due to the limitation of technology, the measurement results of related thermal conductivity for the CMP-gels have not been found. However, the prediction of thermal conductivity for the CMP-gels provides reference and guidance for future technological development. Next, the impact factors effect on this predicted thermal conductivity are discussed in detail in the following section.

### 2.2. Factors Influencing the Equivalent Thermal Conductivity

A parametric study was conducted using the validated model to explore the influence of key structural, material, and environmental parameters on the thermal conductivity components of CMP-gels, specifically using PMMA properties as a base case. The effects of five impact factors on the conductive, radiative and total thermal conductivities are discussed respectively, i.e., porosity (*φ* = 0.123–0.97), cell size (*d*_H_ = 10 nm–100 μm), the material’s refractive index (*n* = 1.49–2.5), temperature (*T* = 280–320 K), and the material’s extinction coefficient (*k* = 0.005–0.1). The optical properties of pure solid PMMA, including its refractive index and extinction coefficient, were adopted from Tsuda’s experimental data [33], as shown in Supporting Information Figure S9a,b. Meanwhile, the thermal conductivity of pure PMMA across different temperatures was determined using Assael’s experimental data [34], as presented in Supporting Information Figure S10. Radiative calculations spanned wavelengths from 2.6 μm to 90 μm using appropriate numerical precision. Additionally, the frequency interval is set as 2 × 10^12^ H_z_ in the subsequent calculations, and incidence angles are 20,000 varying from 0 to 90°, with the details shown in Figure S11a,b of Supporting Information.

#### 2.2.1. Effect of the Cellular Structure

**Figure 2:**
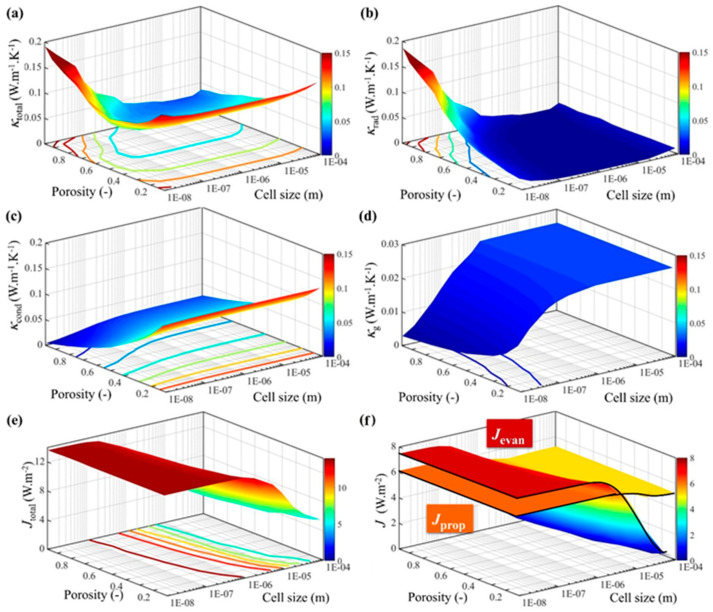
The equivalent thermal conductivity of the CMP-gels versus cell size and porosity: (**a**) the total equivalent thermal conductivity; (**b**) the radiative equivalent thermal conductivity; (**c**) the conductive equivalent thermal conductivity; and (**d**) the gaseous thermal conductivity. The radiant energy flux density versus cell size and porosity: (**e**) the total radiant energy flux density; and (**f**) propagation and the evanescent energy flux density.

To systematically investigate the dependence of thermal conductivity on cell size *d*_H_ and porosity *φ*, we performed calculations for 156 distinct models spanning 10 nm ≤ *d*_H_ ≤ 100 μm and 0.123 ≤ *φ* ≤ 0.97 at *T* = 300 K, with the details shown in Section S1.1 of Supporting Information. The refractive index and extinction coefficient of PMMA were derived from the measurements conducted by Tsuda [33]. The predicted results of the total, radiative, conductive, and gaseous thermal conductivities are illustrated in Figure 2a–d, respectively. Additionally, the computed values for total, propagation, and evanescent radiant energy flux density are presented in Figure 2e,f.

Figure 2a reveals that *κ*_total_ reached its maximum value of 0.192 W·m^−1^·K^−1^ at *d*_H_ = 10 nm and *φ* = 0.97. Within the range of 1 μm ≤ *d*_H_ ≤ 100 μm and 0.74 ≤ *φ* ≤ 0.97, *κ*_total_ exhibited minimum values below 0.03 W·m^−1^·K^−1^. These results break the points in most papers that, due to the Knudsen effect [35,36] will be used to minimize the thermal conductivity when the d_H_ decreases below 100 nm. The reduced cell size leads to an increase in *κ*_total_, primarily due to enhanced *κ*_s_ and *κ*_rad_ contributions [12]. The *κ*_rad_ gradually increased when *φ* increased, as seen in Figure 2b. The results demonstrate that *κ*_rad_ reached its minimum value (≤0.0001 W·m^−1^·K^−1^) when *d*_H_ = 10 μm and *φ* ≤ 0.67. As shown in Figure 2b, *κ*_cond_ exhibited a gradual decrease with increasing *φ* and decreasing *d*_H_. That’s because, when calculating *κ*_cond_, *κ*_g_ accounted for a larger proportion than *κ*_s_, which is consistent with the trend in Figure 2c. In addition, when *φ* ≤ 0.67 and *d*_H_ ≥ 10 μm, the porosity has a greater impact on reducing *κ*_cond_ because the sensitivity to porosity is more pronounced than sensitivity to cell size when *d*_H_ ≥ 10 μm.

In order to elucidate the mechanism underlying the region of minimum radiative thermal conductivity, it is essential to consider the total radiation energy flux density, denoted as *J*_total_, the evanescent energy flux density *J*_evan_ and the propagating energy flux density *J*_prop_ are presented in Figure 2e,f. In Figure 2e,f, as *φ* increased, *J*_total_, *J*_prop_ and *J*_evan_ decreased, while as *d*_H_ increased, *J*_total_, *J*_prop_ and *J*_evan_ increased. Meanwhile, when *d*_H_ ≤ 10 nm, *J*_total_, *J*_prop_ and *J*_evan_ increased slowly as *d*_H_ decreased. However, compared with *J*_evan_, *J*_prop_ did not change obviously with *d*_H_ and *φ*, as it can be seen in Figure 2f.

#### 2.2.2. Effect of Temperature

Since the CMP-gels are mostly applied as insulation materials in our daily lives, without significant temperature changes. Therefore, in this section, the temperatures were discussed as *T* = 280 K, 290 K, 300 K, 310 K, and 320 K, respectively. Ignoring the effect of temperature on the change of thermal conductivity of the pure solid material, the temperature differential for heat transfer between the higher and lower temperature states was established at 1 K, in accordance with the principles of non-equilibrium thermodynamics [37,38,39]. The thermal conductivities of pure PMMA at various temperatures were sourced from the experimental measurements conducted by Assael [34]. Additionally, the refractive index (n) and spectral extinction coefficient (k) of pure PMMA were obtained from the experimental findings of Tsuda [33].

**Figure 3:**
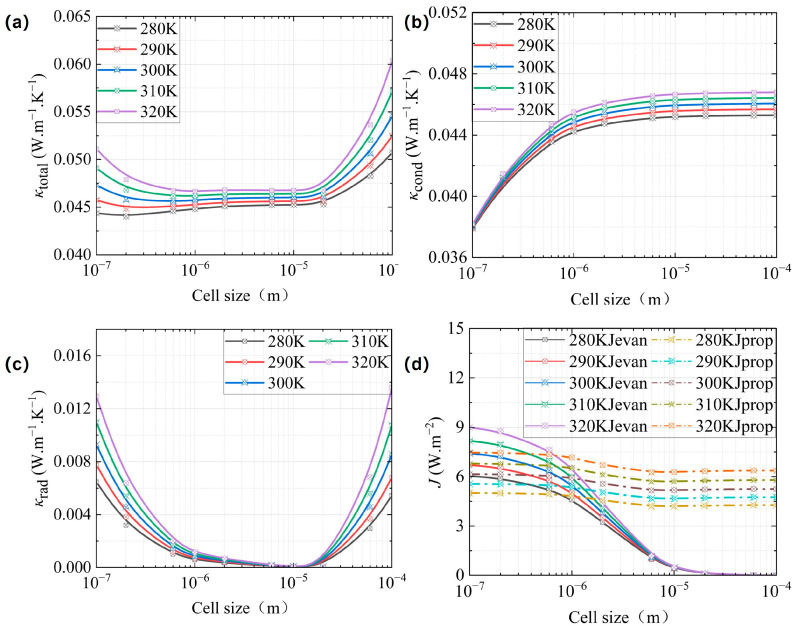
The equivalent thermal conductivity of CMP-gels versus cell size at different temperatures: (**a**) the total equivalent thermal conductivity; (**b**) the conductive equivalent thermal conductivity; and (**c**) the radiative equivalent thermal conductivity. (**d**) The propagation and the evanescent energy flux density versus cell size at different temperatures.

As seen in Figure 3a–c, at a given *φ* = 0.83, the total thermal conductivity, conductive thermal conductivity, and radiative thermal conductivity all increase with temperature. In Figure 3a, the smaller the cell size, *κ*_total_ decreases slightly and then increases with decreasing cell size when *d*_H_ < 2 μm, while the larger the cell size, *κ*_total_ decreases and then levels off with decreasing cell size when *d*_H_ > 2 μm. As illustrated in Figure 3b, there is a positive correlation between cell size and the increase in *κ*_cond_, particularly evident when the *d*_H_ exceeds 500 nm. Conversely, for cell sizes that are smaller, a significant increase in *κ*_rad_ is observed when *d*_H_ is less than 20 μm. However, for cell sizes that fall within the range of 20 μm to 100 μm, a similar trend of increased *κ*_rad_ is noted with larger cell sizes, as depicted in Figure 3c.

In comparison to Figure 3b,c, it is evident that a reduction in cell size results in a more pronounced influence of temperature on *κ*_rad_ relative to *κ*_cond_. Additionally, as illustrated in Figure 3d, an increase in temperature correlates with elevated values of *J*_prop_ and *J*_evan_. *J*_evan_ changed significantly with an increase in *d*_H_ when compared with *J*_prop_. But his change was less apparent when *d*_H_ ≥ 5 μm.

For *κ*_cond_, an increase in temperature resulted in a corresponding rise in both *κ*_s_ and *κ*_g_, as determined by Equations (5) and (6), respectively. By integrating *κ*_s_ and *κ*_g_ into Equation (4), it was observed that *κ*_cond_ exhibited an increase in response to rising temperatures. Conversely, for *κ*_rad_, an increase in temperature was associated with a corresponding rise in radiant energy density, in accordance with Planck’s spectral distribution law [26,40]. Consequently, as determined by Equation (17), the final integrated radiant energy flux density increased, which was consistent with the trend of both *J*_prop_ and *J*_evan_ shown in Figure 3d. On the basis of Equation (18), the increases in both *κ*_cond_ and *κ*_rad_ led to an increase in *κ*_total_.

#### 2.2.3. Effect of Material Properties

In the context of thermal radiation within mesoporous dielectric materials, the thermal conductivity due to radiation, denoted as *κ*_rad_, is associated with the relative permittivity *ε*, which can be expressed as *ε* = (*n*+ i*k*)^2^. Here, *n* represents the refractive index of the material, while *k* signifies the extinction coefficient [41]. In this study, the values of the real component *n*) and the imaginary component (*k*) are treated as independent variables in order to isolate their respective influences. That is when one parameter changes with wavelength while another parameter is unvaried.

**Figure 4:**
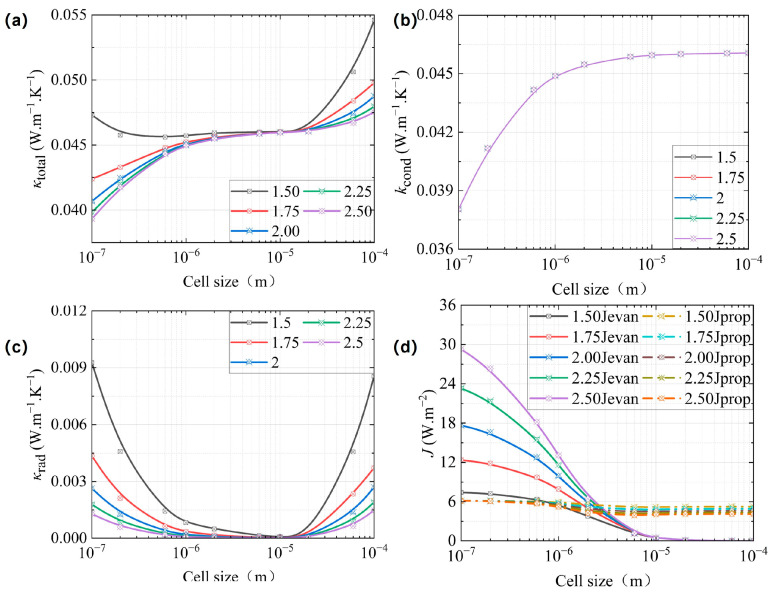
The equivalent thermal conductivity of CMP-gels versus the cell size at different refractive indexes: (**a**) the total equivalent thermal conductivity; (**b**) the conductive equivalent thermal conductivity; and (**c**) the radiative equivalent thermal conductivity. (**d**) The propagation and the evanescent energy flux density versus cell size at different refractive indexes.

The refractive index of dielectric materials, such as polymers, typically falls within the range of 1.4 to 2.0 [42]. For this study, five average refractive index models were selected, specifically *n* = 1.50, 1.75, 2.00, 2.25, and 2.50. The distributions of these indices with respect to wavelength were proportionally adjusted based on Tsuda’s experimental findings [33]. The five models maintained identical parameters: *T* = 300 K, *φ* = 0.83, solid-phase thermal conductivity = 0.19 W·m^−1^·K^−1^, and extinction coefficient from Tsuda’s measurements [33]. The results of this analysis are presented in Figure 4.

In Figure 4a–c that *κ*_total_ and *κ*_rad_ decreased with increasing refractive index *n*, while *κ*_cond_ do not change with increasing refractive index *n*. This is due to the fact that according to the Fresnel equation for reflection, the Fresnel reflection coefficient for p-polarization (*r*_p_) decreases with increasing *n*, leading to a larger angle for total internal reflection, which eventually increases the evanescent energy flux. That is, the evanescent waves would dominate in the total radiant energy flux at a smaller cell size, while propagating waves would dominate at a larger cell size, according to Equations (8) and (9), which is consistent with the trend in Figure 4d. However, as *n* increased, both *σ*_a,λ_ and *σ*_s,λ_ rose according to Equations (15) and (16), consequently increasing *σ*_e,R_. The combined influence of *J*_rad_ and *σ*_e,R_ caused *κ*_rad_ to decrease with *n*. Ultimately, this variation in *κ*_rad_ resulted in an increase in *κ*_total_, as described by Equation (18).

**Figure 5:**
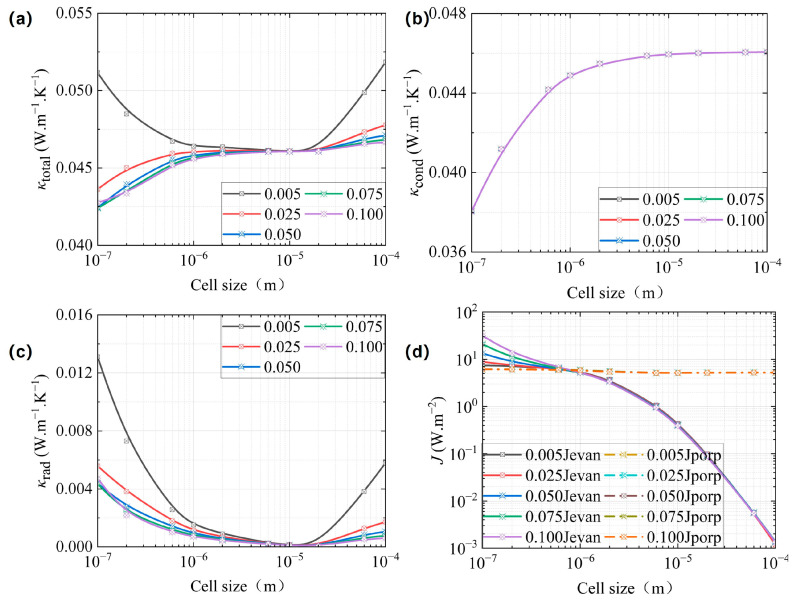
The equivalent thermal conductivity of CMP-gels versus cell size at different extinction coefficients: (**a**) the total equivalent thermal conductivity; (**b**) the conductive equivalent thermal conductivity; and (**c**) the radiative equivalent thermal conductivity. (**d**) The propagation and the evanescent energy flux density versus cell size at different extinction coefficients.

For the dielectric material, the extinction coefficient is much smaller than other materials [43]. This section analyzes five model groups with average extinction coefficients set to *k* = 0.005, 0.025, 0.05, 0.075, and 0.1, where the wavelength-dependent distribution was scaled proportionally based on Tsuda’s experimental data [33]. Additionally, all five model groups shared identical parameters: *T* = 300 K and *φ* = 0.83. The corresponding calculation results are presented in Figure 5.

Figure 5a,c reveal that both *κ*_total_ and *κ*_rad_ decreased with increasing extinction coefficient *k* when *d*_H_ > 200 nm. In contrast, Figure 5b shows the five curves overlapping, which indicates that *κ*_cond_ remained unaffected by variations in *k*. This behavior occurs because the material’s absorption coefficient (*α* = 4π*k*/*λ*) increases with *k* [5]. Consequently, the enhanced absorption coefficient α increased both *σ_a_*_,λ_ and *σ*_e,R_, as determined by Equations (12) and (14). According to Equations (8) and (9), the evanescent radiant energy flux density increased with an increase in *k*, which was consistent with the trend of both *J*_evan_, as shown in Figure 5d, while propagating radiant energy flux density almost have no change with an increase in *k*. Therefore, an increase in *J*_total_ and/or an increase in *σ*_e,R_, led to a varied *κ*_rad_, as calculated by Equation (17). Based on Equation (18), the enhancement of *κ*_rad_ consequently resulted in an increase in *κ*_total_.

## 3. Conclusions

This study developed an innovative fractal-based mathematical model for precise prediction of thermal conductivity in closed mesoporous polymer gels, incorporating both conductive and radiative heat transfer mechanisms across micro- and nanoscale dimensions. The conductive thermal conductivity was modeled using a diagonal cross fractal geometry integrated with Kirchhoff’s law, while radiative thermal conductivity was rigorously derived using comprehensive Rosseland diffusion approximation coupled with Mie scattering theory. The model predictions were validated against experimental results from various mesoporous polymer gel materials, demonstrating excellent agreement with a maximum prediction error of less than 11.2%. A systematic parametric study revealed critical insights into factors influencing thermal conductivity:

The optimal thermal insulation performance occurred within a specific porosity range (0.74–0.97) and cell size range (1–100 µm).

Thermal conductivity exhibited a positive correlation with temperature due to enhanced conductive and radiative heat transfer contributions.

Increasing the refractive index reduced radiative thermal conductivity by promoting internal reflection effects.

Higher extinction coefficients led to decreased radiative thermal conductivity, particularly at cell sizes larger than 200 nm, due to stronger absorption phenomena.

These findings not only offer valuable theoretical guidance for the design and optimization of mesoporous polymer gels as effective thermal insulation materials, but also advance the understanding of microscale heat transfer mechanisms crucial for energy-efficient material engineering and applications.

## 4. Materials and Methods

### 4.1. Geometric Model

**Figure 6:**
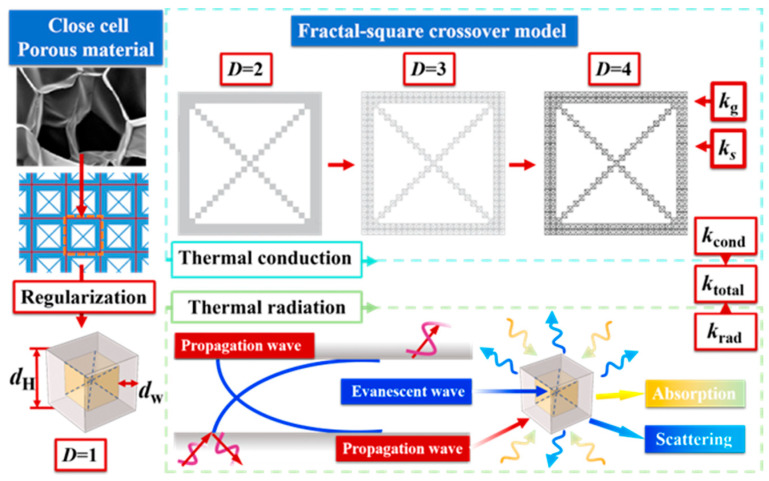
Schematic representation of the heat transfer model for CMP-gels.

The intricate and often disordered structure of porous materials, including polymer gels, frequently exhibits fractal characteristics, meaning statistical self-similarity across different length scales [44,45]. Fractal square models like the Sierpinski gasket [46,47,48] and the Vicsek model [17,49] were used widely in heat transfer research of porous materials. Consequently, in this study, the DCF model was built as thermal conduction model of the CMP-gels, while the unit cube was chosen as the radiative model, as it shown in Figure 6.

### 4.2. The Conductive Equivalent Thermal Conductivity

**Figure 7:**
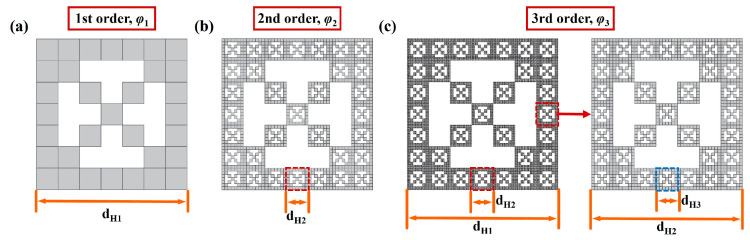
Schematic diagram of the DCF models at different fractal order: (**a**) the first order DCF model; (**b**) the second order DCF model; (**c**) the third order DCF model.

For the thermal conduction model seen in Figure 6., any heat transfer section was chosen and regularized into a two-dimensional third-stage DCF model. In this kind of DCF model, for example, when the porosity *φ* of the CMP-gel is equal to 0.33, the DCF model was established at each fractal stage, as can be seen in Figure 7. In this model, there are (*N* × *N* – *m* × *m* + 2*m* − 1) solid squares and (*m* × *m* − 2*m* + 1) air squares. Specifically, the entire square area is divided into an *N* × *N* grid of smaller squares. Among these, the central region, consisting of (*m* × *m* − 2*m* + 1) smaller squares, is occupied by air holes, effectively ‘holding’ or ‘defining’ the position of these squares. It is important to note that *N* is greater than m, and both *N*, *m*, (*m* + 1)/2, and (*N* − *m*)/2 are positive integers. At this time, a square of size *d*_H1_/*N* is used to measure the DCF pattern, there are (*N* × *N* – *m* × *m* + 2*m* − 1) units. Under this situation, the porosity of the first order DCF model $\varphi_{1}=\frac{m^{2}-2m+1}{N^{2}}$ and the side length of the big square *d*_H1_ were determined, as shown in Figure 7a. Then, each of the remaining (*N* × *N* – *m* × *m* + 2*m* − 1) small squares was also divided into *N* × *N* equal parts, with the air instated the solid material in middle (*m* × *m* − 2*m* + 1) units, and continued infinitely.

**Figure 8:**
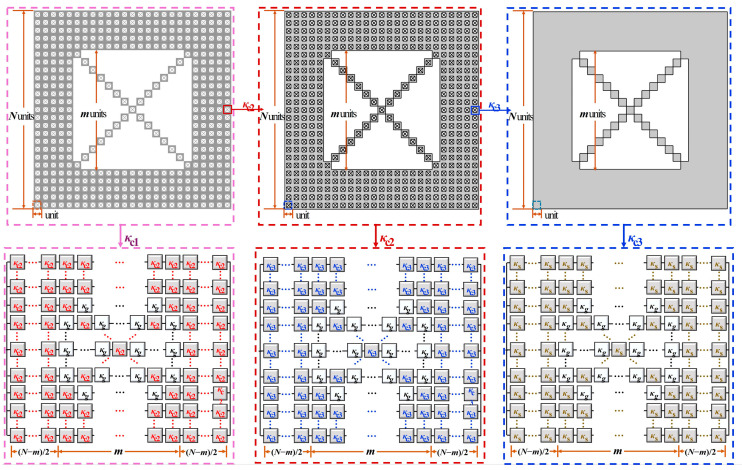
Schematic representation of the equivalent thermal resistance within the DCF model.

As it can be seen in Figure 7b,c, the porosity of the second order DCF model $\varphi_{2}=1-\frac{{\left({N^{2}-m}^{2}+2m-1\right)}^{2}}{N^{4}}$, while the porosity of the third order DCF model $\varphi =\varphi_{3}=1-\frac{{\left({N^{2}-m}^{2}+2m-1\right)}^{4}}{N^{8}}$. In this study, for the convenience of modeling and calculation, thermal conduction models were all chosen, the third order fractal model, and the cell size of the porous material *d*_H_ is defined as the length of unit square *d*_H3_ with meeting the relationship as ${d_{H}=d}_{H3}=\frac{1}{N}d_{H2}=\frac{1}{N^{2}}d_{H1}$ as shown in Figure 7c. Combined with Kirchhoff’s law, the thermal conduction model is equivalent to the thermal resistance series-parallel schematic diagram, as shown in Figure 8.

The thermal conductivity *κ*_c1_ of the entire thermal conduction model can be regarded as composed of (*N* × *N* – *m* × *m* + 2*m* − 1) second order thermal resistances *κ*_c2_ and gaseous thermal resistances *κ*_g_. Similarly, the second order thermal resistance *κ*_c2_ can be seen to be composed of (*N* × *N* – *m* × *m* + 2*m* − 1) unit thermal resistances *κ*_c3_ and gaseous thermal resistance *κ*_g_. Among them, the unit thermal resistance κ_c3_ as the basic unit, can be regarded as composed of (*N* × *N* − *m* × *m* + 2*m* − 1) solid thermal resistances *κ*_s_ and gaseous thermal resistance *κ*_g_. The overall conductive thermal conductivity of the third-order fractal, *κ*_cond_, is derived by considering the series and parallel arrangement of thermal resistances at each fractal level.

(1) $$\kappa_{\mathrm{cond}}=\kappa_{c1}={\left[\frac{\left(N-m\right)}{N\kappa_{c2}}+\frac{\left(m-1\right)}{\left(N-m+2\right)\kappa_{c2}+\left(m-2\right)\kappa_{g}}+\frac{1}{\left(N-m-1\right)\kappa_{c2}+\left(m-1\right)\kappa_{g}}\right]}^{-1}$$

where *κ*_g_ is the conductivity of the gas phase, $\kappa_{c2}$ is the conductive thermal conductivity of the second order fractal square model, obtained from:

(2) $$\kappa_{c2}={\left[\frac{\left(N-m\right)}{N\kappa_{c3}}+\frac{\left(m-1\right)}{\left(N-m+2\right)\kappa_{c3}+\left(m-2\right)\kappa_{g}}+\frac{1}{\left(N-m-1\right)\kappa_{c3}+\left(m-1\right)\kappa_{g}}\right]}^{-1}$$

and the unit conductivity $\kappa_{c3}$ depends on the solid matrix conductivity *κ*_s_ and *κ*_g_:

(3) $$\kappa_{c3}={\left[\frac{\left(N-m\right)}{N\kappa_{s}}+\frac{\left(m-1\right)}{\left(N-m+2\right)\kappa_{s}+\left(m-2\right)\kappa_{g}}+\frac{1}{\left(N-m-1\right)\kappa_{s}+\left(m-1\right)\kappa_{g}}\right]}^{-1}$$

where *κ*_s_ represents the thermal conductivity of the solid phase of the pure material at micro/nanoscale. Then, putting the Equations (2) and (3) into Equation (1), Equation (1) becomes as follows:

(4) $$\kappa_{\mathrm{cond}}=\frac{\sum_{i=1}^{9}C_{i}\kappa_{s}^{10-i}\kappa_{g}^{i-1}}{\sum_{i=10}^{18}C_{i}\kappa_{s}^{18-i}\kappa_{g}^{i-10}}$$

The values of each polynomial coefficient *C*_i_ are in the Supporting Information.

In this work, the solid thermal conduction at the microscale was obtained following Chen’s model [50] as:

(5) $$\kappa_{s}=\frac{0.75d_{w}/\Lambda_{s}}{0.75d_{w}/\Lambda_{s}+1}\kappa_{bulk}$$

where *κ*_bulk_ is the bulk polymer thermal conductivity, $\Lambda_{s}$ is the phonon mean free path in the solid, and the average wall thickness of the unit cell $d_{w}=\frac{\left(1-\varphi^{1/2}\right)}{2}d_{H}$. The gaseous conductivity *κ*_g_ within the confined pores is reduced due to the Knudsen effect [51,52], where gas molecule-wall collisions become significant relative to molecule-molecule collisions. This is modeled as [53]:

(6) $$\kappa_{g}=\frac{\kappa_{g,bulk}}{1+2\beta Κn}$$

where *κ*_g,bulk_ is the bulk gas thermal conductivity (0.026 W·m^−1^·K^−1^ for air at 300 K and 1 bar) [54,55,56], *β* is an energy accommodation coefficient (1.94 for air at 300 K and 1 bar) [53]. *Kn* = $\Lambda_{g}$/*d*_H3_ is the Knudsen number, which refers to the ratio of the mean free path of the air $\Lambda_{g}$(68 nm for air at 300 K and 1 bar) [14] to the cell size *d*_H3_.

According to this fractal construction method, in this study, ten thermal conductive models were built with the different porosity as followed: *φ* = 0.07, 0.16, 0.28, 0.41, 0.54, 0.68, 0.79, 0.89, 0.95 and 0.99, the details of the geometry shapes and parameters are shown in Supporting Information Figure S1. The conductive thermal conductivity of the fractal square model, denoted as *κ*_cond_, was determined using Equation (4).

### 4.3. The Radiative Equivalent Thermal Conductivity

In recent years, researchers have discovered that the thermal radiant energy emitted by two-dimensional microscale objects surpasses the energy predicted by the Planck Stefan-Boltzmann law for blackbody radiation [57,58,59]. Meanwhile, in the porous dielectric material with mesoporous, thermal radiation constitutes a significant portion of the overall thermal conductivity [12]. Therefore, the radiative thermal conductivity of CMP-gels must take into account microscale radiation as delineated by Maxwell’s equations, which govern the propagation of electromagnetic waves and their interactions with matter [60]. Additionally, the integration of fluctuational electrodynamics with the fluctuation-dissipation theorem and Maxwell’s equations [53,61] provides a comprehensive framework for understanding the emission, propagation, and absorption of thermal radiation in both near-field and far-field contexts [62,63].

At the microscale, the net radiative energy flux between parallel surfaces is [62,64]:

(7) $$q_{rad}^{\prime \prime }=\frac{1}{\pi^{2}}\int_{0}^{\infty }d\omega [\Theta \left(\omega ,T_{H}\right)-\Theta \left(\omega ,T_{L}\right)]\int_{0}^{\infty }Z(\omega ,\beta )d\beta$$

The detailed assumptions and derivation process are in the Section S1.2.1 of Supporting Information. In Equation (7), Z(ω,β) is the spectral exchange function, representing the contribution of different wave modes (characterized by the parallel wavevector component (β) to energy transfer [63]. Note that the expression of Z(ω,β) is different for evanescent β>ω∕c and propagating β<ω∕c waves [62],

(8) $$Z_{prop}\left(\omega ,\beta \right)=\frac{\beta (1-p_{01}^{s})(1-p_{02}^{s})}{4{\left|1-r_{01}^{s}r_{02}^{s}e^{i2\gamma_{0}d}\right|}^{2}}+\frac{\beta (1-p_{01}^{p})(1-p_{02}^{p})}{4{\left|1-r_{01}^{p}r_{02}^{p}e^{i2\gamma_{0}d}\right|}^{2}}$$

and

(9) $$Z_{evan}\left(\omega ,\beta \right)=\frac{Im(r_{01}^{s})Im(r_{02}^{s})\beta e^{-2Im(\gamma_{0})d}}{4{\left|1-r_{01}^{s}r_{02}^{s}e^{-2Im(\gamma_{0)}d}\right|}^{2}}+\frac{Im(r_{01}^{p})Im(r_{02}^{p})\beta e^{-2Im(\gamma_{0})d}}{4{\left|1-r_{01}^{p}r_{02}^{p}e^{-2Im(\gamma_{0})d}\right|}^{2}}$$

In Equations (8) and (9), the variable $r_{0j}^{s}=(\gamma_{0}-\gamma_{j})∕(\gamma_{0}+\gamma_{j})$ represents the Fresnel reflection coefficient associated with s-polarization, while $r_{0j}^{p}=(\varepsilon_{j}\gamma_{0}-\gamma_{j})∕(\varepsilon_{j}\gamma_{0}+\gamma_{j})$ denotes the Fresnel reflection coefficient corresponding to p-polarization at the boundary between air and a dielectric medium [63].

In Equation (7), $\Theta \left(\omega ,T\right)$ is the mean energy of a Planck oscillator given by [64,65]:

(10) $$\Theta \left(\omega ,T\right)=\frac{\hbar \omega }{\exp\left(\hbar \omega ∕k_{B}T\right)-1}$$

where ℏ denotes the reduced Planck constant (h/2π), and $k_{B}$ represents the Boltzmann constant [63].

By incorporating Equations (7)–(10) into the Rosseland diffusion approximation [26], the radiant heat flux along the heat transfer direction z can be expressed as:

(11) $$q\left(z\right)=-\frac{4}{3\sigma_{e,R}}\frac{\partial q_{rad}^{\prime \prime }}{\partial z}$$

where *σ*_e,R_ denotes the Rosseland average extinction coefficient, calculated according to [64,66]:

(12) $$\frac{1}{\sigma_{e,R}}=\frac{\int_{0}^{\infty }\frac{1}{\sigma_{e,\lambda }}f\left(\lambda ,T\right)d\lambda }{\int_{0}^{\infty }f\left(\lambda ,T\right)d\lambda }$$

where λ represents the wavelength, *σ*_e,λ_ denotes the spectral extinction coefficient, and f(λ,T) stands for the spectral distribution of Planck blackbody emission as defined in [11,53]

(13) $$f\left(\lambda ,T\right)=\frac{\partial e_{b,\lambda }}{\partial T}=\frac{C_{1}}{\lambda^{5}(e^{C_{2}/\lambda T}-1)}$$

where $e_{b,\lambda }$ denotes the blackbody spectral intensity, $C_{1}=2\pi hc^{2}$ represents the first radiation constant, $C_{2}=hc/k_{B}$ corresponds to the second radiation constant, and h signifies the Planck constant [49].

In Equation (12), for a uniform isotropic medium, the spectral extinction coefficient *σ*_e,λ_ equals the sum of the absorption coefficient *σ*_a,λ_ and scattering coefficient *σ*_s,λ_ [26,67]:

(14) $$\sigma_{e,\lambda }=\sigma_{a,\lambda }+\sigma_{s,\lambda }$$

The calculation and verification showed that the cellular shapes and the surface area have no obvious influence on the radiative thermal conductivity, as shown in Section S1.2.2 of Supporting Information. Therefore, for ease of calculation, the cellular shape can be equivalently transformed into a sphere at the same volume. For the spherical and monodispersed closed cell pores, where porosity plays an important role, the scattering characteristic satisfies the Mie scattering [68]. Therefore, for the transparent porous materials, the *σ*_a,λ_ and the *σ*_s,λ_ can be calculated by Mie theory as follows [68]:

(15) $${\sigma_{a,\lambda }=N}_{v}{\pi r_{p}}^{2}Q_{a}\left(r,\lambda ,n\left(\lambda \right)\right)$$

(16) $${\sigma_{s,\lambda }=N}_{v}{\pi r_{p}}^{2}Q_{s}\left(r,\lambda ,n\left(\lambda \right)\right)$$

where *r*_p_ is the equivalent radius of air pore $\;r_{p}={\left(\frac{3\varphi }{4\pi }\right)}^{1/3}d_{H}$, *N*_v_ is the density of pores found in the material, calculated by $N_{v}=P/(0.75{\pi r_{p}}^{3})$, where the scattering phase function *P* for mono-dispersion particles, which describes the angle dependent scattering of the incident beam, is defined by [12]: *P* = 4*πσ_λ_*_,*θ*_/*σ_λ_*. In Equations (15) and (16), *Q_s_* and *Q_a_* are the scattering efficiency and absorption efficiency calculated by Mie theory as functions of the particle size, wavelength, and the refractive index of solid material [31].

The radiative equivalent thermal conductivity, defined through Fourier’s law, can be expressed using the Rosseland diffusion approximation [12] by combining Equation (7) with Equation (11):

(17) $$\kappa_{rad}=\frac{4\sigma_{SB}}{3\pi^{2}\sigma_{e,R}}\frac{\partial (\int_{0}^{\infty }d\omega [\Theta \left(\omega ,T_{1}\right)-\Theta \left(\omega ,T_{2}\right)]\int_{0}^{\infty }Z(\omega ,\beta )d\beta }{\partial T}$$

The radiative thermal conductivity of CMP-gels was calculated using this formulation.

### 4.4. The Total Equivalent Thermal Conductivity

**Figure 9:**
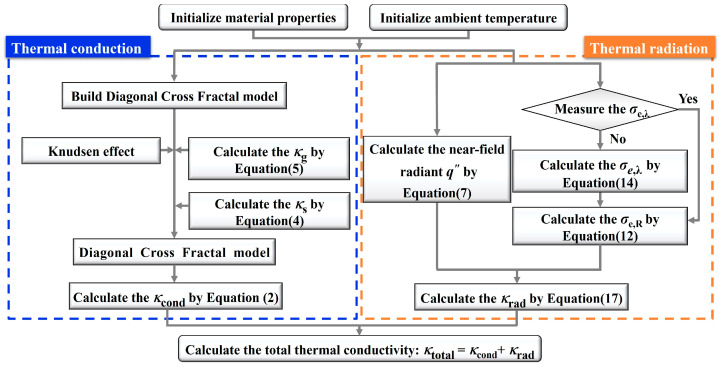
Flow diagram of the equivalent thermal conductivity calculation progress.

It is important to highlight that thermal convection has a limited influence in porous closed-cell materials characterized by pore diameters less than 4 mm [69]. Therefore, this research indicates that the heat transfer mechanisms in CMP-gels primarily consist of two components: thermal conduction and thermal radiation. In contrast, thermal convection is considered negligible at this confined scale [6,7]. Assuming conduction and radiation are the dominant heat transfer modes and act in parallel, the total effective thermal conductivity (ETC) *κ*_total_ is the sum of the two components [5]:

(18) $$\kappa_{total}=\kappa_{cond}+\kappa_{rad}$$

where *κ*_cond_ is calculated from the DCF model from Equation (4) and *κ*_rad_ is calculated using the microscale-informed Rosseland approximation from Equation (17). The overall calculation procedure is summarized in Figure 9.

## Data Availability

The original contributions presented in this study are included in the article. Further inquiries can be directed to the corresponding authors.

## Supplementary Materials

The following supporting information can be downloaded at: https://www.mdpi.com/article/10.3390/gels11060391/s1, Figure S1. The fractal-square crossover models with different *φ*: (a) *φ* = 0.07; (b) *φ* = 0.16; (c) *φ* = 0.28; (d) *φ* = 0.41; (e) *φ* = 0.54; (f) *φ* = 0.68; (g) *φ* = 0.79; (h) *φ* = 0.89; (i) *φ* = 0.95; (j) *φ* = 0.99. Figure S2. Schematic illustration of near field radiative heat transfer between two close dielectric skeletons, at temperatures *T*_H_ and *T*_L_, separated by an air pore *d*_p_. Figure S3. Schematic diagram of Yee cell grid structure. Figure S4. Magnetic field strength and electric field direction at the z-direction cross section of different models at different wavelength: (a) *d*_H_ = 5 μm, *λ* = 5 μm; (b) *d*_H_ = 5 μm, *λ* = 10 μm; (c) *d*_H_ = 5 μm, *λ* = 20 μm; (d) *d*_H_ = 10 μm, *λ* = 5 μm; (e) *d*_H_ = 10 μm, *λ* = 10 μm; (f) *d*_H_ = 10 μm, *λ* = 20 μm; (g) *d*_H_ = 20 μm, *λ* = 5 μm; (h) *d*_H_ = 20 μm, *λ* = 10 μm; and (i) *d*_H_ = 20 μm, *λ* = 20 μm. Figure S5. Schematic diagram of the different pore shape: (a) S4 model; (b) S6 model; (c) S8 model; (d) SC model. Figure S6. The thermal conductivities versus cell size at different pore shapes: (a) the total thermal conductivity; (b) the conductive thermal conductivity; (c) the radiative thermal conductivity. Figure S7. Schematic diagram of the (a) 1.0× model; (b) 1.25× model, (c) 1.5× model, (d) 1.75× model; and (e) 2.0× model. Figure S8. The thermal conductivity versus cell size at different surface areas: (a) the total thermal conductivity; (b) the conductive thermal conductivity; (c) the radiative thermal conductivity. Figure S9. (a) The refractive index of PMMA; (b) the extinction coefficient of PMMA. Figure S10. The thermal conductivity of PMMA with different temperature. Figure S11. (a) The effect of frequency interval on net radiant energy flux density; (b) the effect of theta points on net radiant energy flux density. Figure S12. (a) The effect of temperature interval on net radiant energy flux density; (b) the effect of temperature interval on radiative thermal conductivity. Table S1. The geometry parameters *N* and *m* of fractal-square crossover model at different *φ*. Table S2. Structural parameters of surface models with different volume ratios.

## Nomenclature

*A*	cross-sectional area	m
*c*	speed of light in vacuum	m·s^−1^
*d*	thickness	m
*E*	electric field vector	V·m^−1^
*G*	dyadic Green function	m^−1^
*H*	magnetic field vector	A·m^−1^
*ħ*	Planck constant divided by 2π	J·s
*J*	energy flux density	W·m^−2^
*k*	material extinction coefficient	-
*k*_B_	Boltzmann constant	J·K^−1^
*m*	numbers of small air square	-
*n*	refractive index	-
***n***	normal vector	-
*N*	particle numbers unit numbers of small solid square	-
*P*	scattering phase function	-
*r*	radius of polymer particles/Fresnel reflection coefficient	m/-
*S*	specific surface area	m^2^·kg^−1^
*T*	temperature	K
*t*	Fresnel transmission coefficient	-
*q*″	heat flux	W·m^−2^
*x,y,z*	coordinate direction	-
*Θ*	mean energy of the Planck oscillator	J
*κ*	thermal conductivity	W·m^−1^·K^−1^
*ρ*	density	kg·m^−3^
*ε*	relative permittivity	F·m^−1^
*α*	material absorption coefficient	-
*β*	energy interaction between gaseous molecules and the solid surface	-
*σ*	the coefficient	-
*σ*_SB_	Steven-Boltzmann constant.	-
*λ*	wavelength	m
*φ*	porosity	-
*μ*	the permeability	H·m^−1^
*ω*	angular frequency	rad·s^−1^
*Λ*	phonon mean free path	m
**Subscript**		
a	absorption	
bulk	dielectric skeleton	
cond	thermal conduction	
e	extinction	
evan	evanescent wave	
g	gas	
H	cell size	
mp	maximum emissive power peak	
p	equivalent pore size	
prop	propagating wave	
rad	thermal radiation	
s	solid/scattering	
total	total heat transfer	
w	equivalent wall size	
z	z direction	
**Superscript**		
s	s polarization	
p	p polarization	
