# Supplementary material for: Fractal-Based Thermal Conductivity Prediction Modeling for Closed Mesoporous Polymer Gels
Source: Gels. 2025 May 26;11(6):391. doi: 10.3390/gels11060391 (PMC12192145; doi:10.3390/gels11060391)
Supplement: Supplementary file 1 [file gels-11-00391-s001.zip › gels-3633047-supplementary.pdf]

Supporting Information

A Novel Method to Calculate the Thermal Conductivity of Micro/Nano Closed Cell Porous Dielectric Materials

Haiyan Yu [a], Mingdong Li [a], Ning Guo [a], Anqi Chen [a], Xiaohan Ren [a, *], and Haochun Zhang[b, *]

[a] Institute of the Thermal Science and Technology, Shandong University, Jinan 250061, China

[b] School of Energy Science and Engineering, Harbin Institute of Technology, Harbin 150001, China

* Corresponding Authors: renxh@sdu.edu.cn; hczhang@hit.edu.cn

Keywords: Thermal conductivity; Micro/nanoscale thermal radiation; Microscale porous closed cell material; Nanoscale porous closed cell material; Energy management

Contents

S1. Computation Model 2

S1.1 Thermal Conduction Model 2

S1.2 Thermal Radiation Model 4

S1.2.1 Radiant energy calculation 4

S1.2.2 Extinction coefficent calculation 6

The Effect of Cellular Shapes 10

The Effect of Surface Aera 11

S2. Settings of the Materials' Properties 13

S2.1 Materials' Refractive Index and Extinction Coefficient 13

S2.2 Materials' Thermal Conductivities 13

S3. Independence Verification 14

S3.1 Frequency Interval and Spatial Angular Interval 14

S3.2 Temperature Interval 15

References 17

S1. Computation Model

S1.1 Thermal Conduction Model

In Section 2.2 of the main text, the third order fractal-square crossover model was chosen as thermal conduction model, which was built by (*N**N- m*m+2m-1*) small solid squares and the (*m*m-2m+1*) small air squares for each order.

That is, the square was divided into *N*N* small squares, there are (*N*N-m*m+2m-1*) solid squares and (*m*m-2m+1*) air squares., where the *N* and *m* are the positive integer, $N > m$, and both *N*, *m*, (*m+1*)*/2*, and (*N-m*)*/2* are positive integers.. At this time, a square of size d_{H1}/N is used to measure the fractal square pattern, there has (*N*N - m*m+2m-1*) units. Then, each of the remaining (*N*N - m*m+2m-1*) small squares was also divided into *N*N* equal parts, with the air instated the solid material in middle (*m*m-2m+1*) units, and continue infinitely, as shown in Fig.3 in main text. According to this fractal construction method, the porosity of the porous dielectric materials φ is equal to the porosity of the third order fractal model φ_3 as $\varphi = \varphi_3 = 1 - \frac{(N^2-m^2+2m-1\ \ )^4}{N^8}$.

Specifically, in the process of calculating κ_{cond}, the values of each polynomial coefficient C are as follows:

$C_1 = N^7 - (N - m)^7$

$C_2 = N^5m(6N^4 - 12N^3m + 8N^2m^2 - 2Nm^3 + N^2 - Nm + m^2)(N - m)^4$

$C_3 = N^4m^2(15N^6 - 47N^5m + 63N^4m^2 - 44N^3m^3 + 17N^2m^4 - 3Nm^5 - m^6 + 3N^5 - 11N^4m + 16N^3m^2 - 13N^2m^3 + 5Nm^4 + 3N^4 - 6N^3m + 7N^2m^2 - 4Nm^3 + m^4)\ (N - m)^2$

$C_4 = N^3m^3(N - m)(23N^7 - 82N^6m + 137N^5m^2 - 126N^4m^3 + 74N^3m^4 - 24N^2m^5 + 2Nm^6 + m^7 + 9N^6 - 33N^5m + 51N^4m^2 - 47N^3m^3 + 23N^2m^4 - 5Nm^5 + 3N^5 - 7N^4m + 9N^3m^2 - 6N^2m^3 + 2Nm^4)$

$C_5 = 18N^4m^{10} + (-77N^5 - 10N^4)\ m^9 + (172N^6 + 36N^5 + N^4)m^8 + (-235N^7 - 63N^6 - 3N^5)m^7 + (207N^8 + 62N^7 + 4N^6)m6 + (-105N^9 - 36N^8 - 3N^7)m^5 + 25(N^2 + 9/25N + 1/25)N^8m^4$

$C_6 = m^5N^3(18N^6 - 71N^5m + 129N^4m^2 - 134N^3m^3 + 85N^2m^4 - 31Nm^5 + 4m^6 + 3N^5 - 11N^4m + 16N^3m^2 - 13N^2m^3 + 5Nm^4)$

$C_7 = 7(N^3 - 13/7N^2m + 11/7Nm^2 - 4/7m^3)m^6(N - m)^2N^3$

$C_8 = N^3m^7(N - m)^4$

$C_9 = N^4(N^2 - Nm + m^2)^2(N - m)^4$

$C_{10}= m(N^2 - Nm + m^2)^2(N - m)^2(4N^3m^2 - 5N^2m^3 + Nm^4 + 3N^4 - 8N^3m + 5N^2m^2 + 3N^3 - 3N^2m + Nm^2 + N - m)N^2$

$C_{11}= 3m^2(N^2 - Nm + m^2)^2\{N^6 + [-(11m)/3 + 3]N^5 + (9m^2 - 8m + 1)N^4 + (-8m^3 + 6m^2 - 4/3m + 2)N^3 + (3m^4 - 5/3m^3 + 10/3m^2 - 16/3m)N^2 - m^2(m^3 + 10m - 10)N/3 + (2m^4)/3\}(N - m)N$

$C_{12}= (10N^9 - 48N^8m + 117N^7m^2 - 167N^6m^3 + 170N^5m^4 - 129N^4m^5 + 79N^3m^6 - 38N^2m^7 + 10Nm^8 - m^9 + 9N^8 - 36N^7m + 86N^6m^2 - 157N^5m^3 + 186N^4m^4 - 146N^3m^5 + 66N^2m^6 - 10Nm^7 + 16N^7 - 79N^6m + 165N^5m^2 - 192N^4m^3 + 133N^3m^4 - 47N^2m^5 + 4Nm^6)m^3(N^2 - Nm + m^2)$

$C_{13}= 12m^4(N^2 - Nm + m^2)(N - m)\{N^7 + (-13m/4 + 7/4)N^6 + (22/3m^2 - 43/6m + 1/6)N^5 + (-21/2m^3 + 37/3m^2 - 2/3m)N^4 + (125/12m^4 - 49/4m^3 + m^2)N^3 – [41m^3(m^2 - m + 4/41)N^2]/6 + (5/2m^6 - 5/3m^5 + 1/6m^4)N - m^7/3\}$

$C_{14}= (15N^5 - 42N^4m + 65N^3m^2 - 63N^2m^3 + 32Nm^4 - 6m^5 + 6N^4 - 22N^3m + 26N^2m^2 - 10Nm^3) m^5 (N^2 - Nm + m^2)(N - m)^2$

$C_{15}= m^6(N^2 - Nm + m^2)(N - m)^4(7N^2 - 10Nm + 4m^2)$

$C_{16}= m^7(N - m)^7$

$C_{17}=N^2(N^{10}-8N^9m+28N^8m^2-56N^7m^3+70N^6m^4-56N^5m^5+28N^4m^6-8N^3m^7+N^2m^8+12N^9-84N^8m+252N^7m^2-420N^6m^3+420N^5m^4-252N^4m^5+84N^3m^6-12N^2m^7+62N^8-372N^7m+930N^6m^2-1240N^5m^3+930N^4m^4-372N^3m^5+62N^2m^6+180N^7-900N^6m+1800N^5m^2-1800N^4m^3+900N^3m^4-180N^2m^5+321N^6-1284N^5m+1926N^4m^2-1284N^3m^3+321N^2m^4+360N^5-1080N^4m+1080N^3m^2-360N^2m^3+248N^4-496N^3m+248N^2m^2+96N^3-96N^2m+16N^2)$

$C_{18}=N^2(8N^9m-56N^8m^2+170N^7m^3-292N^6m^4+310N^5m^5-208N^4m^6+86N^3m^7-20N^2m^8+2Nm^9-12N^9+168N^8m-763N^7m^2+1739N^6m^3-2290N^5m^4+1822N^4m^5-863N^3m^6+223N^2m^7-24Nm^8-124N^8+1124N^7m-3825N^6m^2+6664N^5m^3-6546N^4m^4+3648N^3m^5-1065N^2m^6+124Nm^7-543N^7+3680N^6m-9543N^5m^2+12339N^4m^3-8414N^3m^4+2841N^2m^5-360Nm^6-1306N^6+6722N^5m-12865N^4m^2+11430N^3m^3-4623N^2m^4+642Nm^5-1863N^5+7034N^4m-9143N^3m^2+4692N^2m^3-720Nm^4-1576N^4+3988N^3m-2896N^2m^2+496Nm^3-732N^3+992N^2m-192Nm^2-144N^2+32Nm)$

In this study, ten thermal conductive models with different porosities were built with the different porosity as followed: φ = 0.07, 0.16, 0.28, 0.41, 0.54, 0.68, 0.79, 0.89, 0.95 and 0.99, as shown in Fig.S1. Meanwhile, the parameters N and m of each fractal-square crossover model are shown in Table S1. It should note that, for the same porosity φ but different cell size d_H, the geometry shapes and parameters N and m of fractal model are the same, while the length of the sides of the square is different.

Table S1. The geometry parameters *N* and *m* of fractal-square crossover model at different *φ*.

ϕ	0.07	0.16	0.28	0.41	0.54	0.68	0.79	0.89	0.95	0.99
N	25	25	25	25	25	25	25	25	25	25
m	5	7	9	11	13	15	17	19	21	23

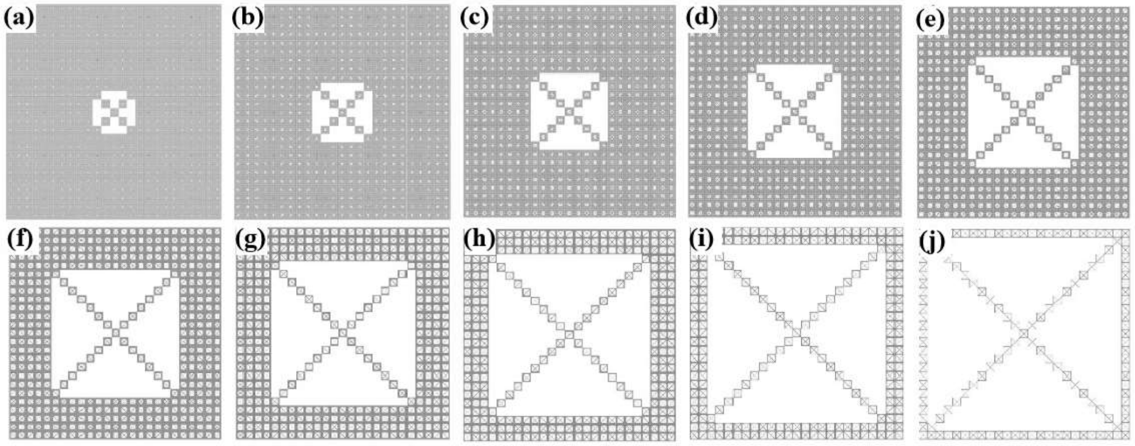

Figure S1. The fractal-square crossover models with different *φ*: (a) *φ* = 0.07; (b) *φ* = 0.16; (c) *φ* = 0.28; (d) *φ* = 0.41; (e) *φ* = 0.54; (f) *φ* = 0.68; (g) *φ* = 0.79; (h) *φ* = 0.89; (i) *φ* = 0.95; (j) *φ* = 0.99.

S1.2 Thermal Radiation Model

S1.2.1 Radiant energy calculation

Since Chen's group [S1, S2] found that the radiant energy of two very closely located objects is much larger than predicted by the Planck Stefan-Boltzman law [S2, S3], the thermal radiation process needs to consider microscale radiative heat transfer. The Maxwell's equations describe the propagation of electromagnetic waves and their interactions with matter [S4, S5]. Furthermore, the fluctuational electrodynamics combining the fluctuation-dissipation theorem with Maxwell's equations [S6, S7] fully describe the emission, propagation and absorption of thermal radiation in both the near and far fields [S8].

A micro/nano-scaled air pore in the porous dielectric material can be simplified into two closely spaced semi-infinite dielectric plates separated by an air gap of width d_p at temperatures T_H and T_L, respectively, as illustrated in Fig. 2. Here it is

assumed that the material is uniform and isotropic. Also, it is assumed that T_H is the high temperature and T_L is the ambient temperature. It is further assumed that the surface of each plate is smooth enough. Furthermore, the radiative heat transfer from the lower temperature plate to the higher temperature plate must also be considered [S9].

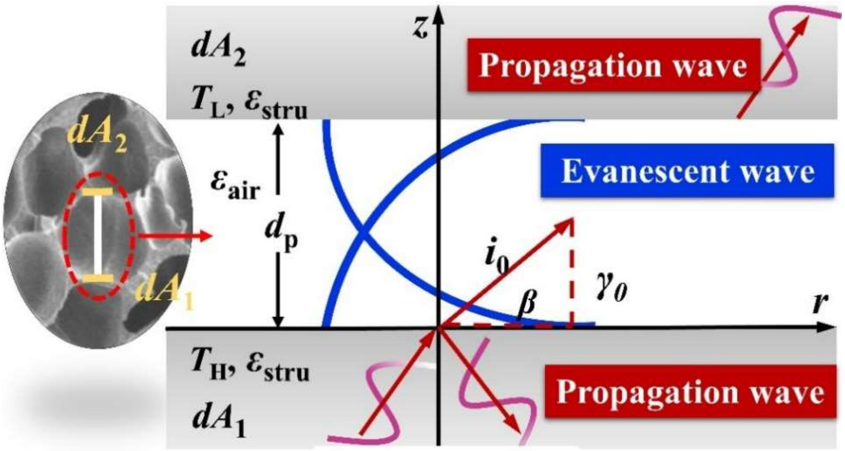

Figure S2. Schematic illustration of near field radiative heat transfer between two close dielectric skeletons, at temperatures T_H and T_L, separated by an air pore d_p.

As shown in Fig. S2, the space variables, x, z, and r, in the cylindrical coordinate system satisfy $x = z + r$, where the z direction is perpendicular to the interface and the r direction is parallel to the interface. The γ_j and β refer to the z component and r component of the wavevector i_j, respectively, for j = 0, 1 and 2. Therefore, $i_0 = \beta\hat{r} + \gamma_0\hat{z}$. The magnitude of i_j is related to the relative permittivity ε_j by $i_0 = \omega / c$ and $i_1 = i_2 = \sqrt{\varepsilon_s}\omega / c$, with ε_g and ε_s being the relative permittivity of air and dielectric skeleton, respectively, and c is the speed of light in vacuum. The dyadic Green's function, $\bar{\bar{G}}(x, x', \omega)$ is essentially a spatial transfer function between a current source j at a location x' and the resultant electric field E at x [S9, S10]. The $\bar{\bar{G}}(x, x', \omega)$ depends on the geometry of the physical system and, for two semi-infinite media shown in Fig. S2, it takes the following form [S11]:

$$\bar{\bar{G}}(x, x', \omega) = \int_0^\infty \frac{i}{4\pi\gamma_j}\left(\hat{s}t_{12}^s\hat{s} + \hat{p}_2 t_{12}^p \hat{p}_1\right)e^{i(\gamma_2 z - \gamma_1 z)}e^{i\beta(r-r')}\beta d\beta \qquad \text{(S1)}$$

where $x = r\hat{r} + z\hat{z}$, $x = r'\hat{r} + z'\hat{z}$, t_{12}^s is the transmission coefficient for s polarization and t_{12}^p is the transmission coefficient for p polarization from medium 1 to medium 2, which are given by Airy's formula [S8, S12]. The unit vectors are $\hat{s} = \hat{r} \times \hat{z}$, $\hat{p}_1 = (\beta\hat{z} - \gamma_1\hat{r}) / i_1$, and $\hat{p}_2 = (\beta\hat{z} - \gamma_2\hat{r}) / i_2$.

S1.2.2 Extinction coefficent calculation

In Section 2.3 of the main text, for a uniform and isotropic material, the extinction coefficient $\sigma_{e,\lambda}$ can also be calculated by summing the absorption coefficient $\sigma_{a,\lambda}$ and the scattering coefficient $\sigma_{s,\lambda}$ [S13, S14]:

$$\sigma_{e,\lambda} = \sigma_{a,\lambda} + \sigma_{s,\lambda} \qquad \text{(S2)}$$

In order to study the effect of cellular shapes and surface area on thermal conductivity, the the absorption coefficient $\sigma_{a,\lambda}$ and the scattering coefficient $\sigma_{s,\lambda}$ could be calculated as follows:

$$\sigma_{a,\lambda} = \frac{-Re \iint_{\Sigma}\{E^{(i)}\times H^{(s)*} + E^{(s)}\times(H^{(i)*} - H^{(s)*})\}\cdot \boldsymbol{n} dA}{N\cdot Re\{E^{(i)}\times H^{(i)*}\}} \qquad \text{(S3)}$$

$$\sigma_{s,\lambda} = \frac{Re \iint_{\Sigma}\{E^{(s)}\times H^{(s)*}\}\cdot \boldsymbol{n} dA}{N\cdot Re\{E^{(i)}\times H^{(i)*}\}} \qquad \text{(S4)}$$

where $E^{(s)}$ and $H^{(s)}$ represent the scattering electric field and the scattering magnetic field, respectively, $E^{(i)}$ and $H^{(i)}$ represent the incident electric field and incident magnetic field, respectively, $\boldsymbol{n}$ is the normal vector of the surface area, A is the unit cross-sectional area and N is the number of cells per unit volume. The $E^{(s)}$, $E^{(i)}$, $H^{(s)}$ and $H^{(i)}$ can be obtained by the time-domain finite-difference (FDTD) method [S15, S16]

The basic theory of Maxwell's equations [S17, S18] can govern the distribution of electromagnetic phenomena, whose curl equations and constitutive equations are written as [S16, S19]:

$$\begin{cases} \nabla\times \boldsymbol{H} = \dfrac{\partial \boldsymbol{D}}{\partial t} + \boldsymbol{J} \\ \nabla\times \boldsymbol{E} = -\dfrac{\partial \boldsymbol{B}}{\partial t} - \boldsymbol{M} \\ \nabla\cdot \boldsymbol{B} = \rho_{\mathrm{e}} \\ \nabla\cdot \boldsymbol{D} = \rho_{\mathrm{m}} \end{cases} \qquad \text{(S5)}$$

where $\boldsymbol{H}$ is the magnetic field intensity, V/m; $\boldsymbol{E}$ is the electric field intensity, $\boldsymbol{B}$ is the magnetic flux density, $\boldsymbol{D}$ is the electric displacement vector, $\boldsymbol{J}$ is the current density, t is the time, and ρ is the volume charge density. The first two equations in Eq. (S5) describe respectively the interaction of the electric field intensity and the magnetic flux density in a vacuum. The material equations are given as [S20, S21]:

$$\begin{cases} \boldsymbol{D} = \varepsilon \boldsymbol{E} \\ \boldsymbol{B} = \mu \boldsymbol{H} \\ \boldsymbol{J} = \sigma \boldsymbol{E} \end{cases} \qquad \text{(S6)}$$

where ε is the permittivity, F/m, μ is the permeability, H/m, and σ is the conductivity, S/m. Finally, the Maxwell equations

in media are obtained by inserting Eq. (S6) into Eq. (S5) and given by [S22]:

$$\begin{cases} \nabla\times\boldsymbol{H}=\varepsilon\dfrac{\partial\boldsymbol{E}}{\partial t}+\sigma\boldsymbol{E} \\ \nabla\times\boldsymbol{E}=-\mu\dfrac{\partial\boldsymbol{H}}{\partial t} \\ \nabla\cdot\boldsymbol{H}=0 \\ \nabla\cdot\boldsymbol{E}=\dfrac{\rho}{\varepsilon} \end{cases} \tag{S7}$$

In three-dimensional cartesian coordinate system, these equations can be written as six scalar equations [S16]:

$$\begin{cases} \dfrac{\partial H_{\mathrm{x}}}{\partial t}=\dfrac{1}{\mu_{\mathrm{x}}}\left(\dfrac{\partial E_{\mathrm{y}}}{\partial z}-\dfrac{\partial E_{\mathrm{z}}}{\partial y}-\sigma_{\mathrm{x}}^{\mathrm{m}}H_{\mathrm{x}}\right) \\ \dfrac{\partial H_{\mathrm{y}}}{\partial t}=\dfrac{1}{\mu_{\mathrm{y}}}\left(\dfrac{\partial E_{\mathrm{z}}}{\partial x}-\dfrac{\partial E_{\mathrm{x}}}{\partial z}-\sigma_{\mathrm{y}}^{\mathrm{m}}H_{\mathrm{y}}\right) \\ \dfrac{\partial H_{\mathrm{z}}}{\partial t}=\dfrac{1}{\mu_{\mathrm{z}}}\left(\dfrac{\partial E_{\mathrm{x}}}{\partial y}-\dfrac{\partial E_{\mathrm{y}}}{\partial z}-\sigma_{\mathrm{z}}^{\mathrm{m}}H_{\mathrm{z}}\right) \\ \dfrac{\partial E_{\mathrm{x}}}{\partial t}=\dfrac{1}{\varepsilon_{\mathrm{x}}}\left(\dfrac{\partial H_{\mathrm{z}}}{\partial y}-\dfrac{\partial H_{\mathrm{y}}}{\partial z}-\sigma_{\mathrm{x}}^{\mathrm{e}}E_{\mathrm{x}}\right) \\ \dfrac{\partial E_{\mathrm{y}}}{\partial t}=\dfrac{1}{\varepsilon_{\mathrm{y}}}\left(\dfrac{\partial H_{\mathrm{x}}}{\partial z}-\dfrac{\partial H_{\mathrm{z}}}{\partial x}-\sigma_{\mathrm{y}}^{\mathrm{e}}E_{\mathrm{y}}\right) \\ \dfrac{\partial E_{\mathrm{z}}}{\partial t}=\dfrac{1}{\varepsilon_{\mathrm{z}}}\left(\dfrac{\partial H_{\mathrm{y}}}{\partial x}-\dfrac{\partial H_{\mathrm{x}}}{\partial y}-\sigma_{\mathrm{z}}^{\mathrm{e}}E_{\mathrm{z}}\right) \end{cases} \tag{S8}$$

where *x*, *y* and *z* are the axis in the Cartesian coordinate system Eq. (S8) establishes the relationship between the Maxwell's equations and the physical model media. During the calculation, the inlet and the outlet are set as the perfectly matched layer boundary conditions. Eq. (S8) can be discretised in 3D using the FDTD method [S23, S24]. Here, the discretization form of the electromagnetic field in the x direction in Cartesian coordinates is given by [S25, S26]:

$$\begin{aligned} E_{\mathrm{x}}^{\mathrm{n}+1}(i+0.5,j,k)=CA(m)\cdot E_{\mathrm{x}}^{\mathrm{n}}(i+0.5,j,k)+ \\ CB(m)\cdot\left[\frac{H_{\mathrm{z}}^{\mathrm{n}+0.5}(i+0.5,j+0.5,k)-H_{\mathrm{z}}^{\mathrm{n}+0.5}(i+0.5,j-0.5,k)}{\Delta y}\right. \\ \left.-\frac{H_{\mathrm{y}}^{\mathrm{n}+0.5}(i+0.5,j,k+0.5)-H_{\mathrm{y}}^{\mathrm{n}+0.5}(i+0.5,j,k-0.5)}{\Delta z}\right] \end{aligned} \tag{S9}$$

$$H_{\mathrm{x}}^{\mathrm{n+1}}\left(i,j+0.5,k+0.5\right)=E_{\mathrm{x}}^{\mathrm{n\text{-}0.5}}\left(i,j+0.5,k+0.5\right)-\frac{\Delta t}{\mu(m)}\cdot\left[\frac{E_{\mathrm{z}}^{\mathrm{n}}\left(i,j+0.5,k+0.5\right)-E_{\mathrm{z}}^{\mathrm{n}}\left(i,j,k+0.5\right)}{\Delta y}-\frac{E_{\mathrm{y}}^{\mathrm{n}}\left(i,j+0.5,k+1\right)-E_{\mathrm{y}}^{\mathrm{n}}\left(i,j+0.5,k\right)}{\Delta z}\right] \tag{S10}$$

$$CA\left(m\right)=\frac{\frac{\varepsilon\left(m\right)}{\Delta t}-\frac{\sigma\left(m\right)}{2}}{\frac{\varepsilon\left(m\right)}{\Delta t}+\frac{\sigma\left(m\right)}{2}},\qquad CB\left(m\right)=\frac{1}{\frac{\varepsilon\left(m\right)}{\Delta t}+\frac{\sigma\left(m\right)}{2}} \tag{S11}$$

where *n* is the calculating steps; *i, j, and k* are the nodes of mesh, the value is integer, and *m* = (*i+0.5, j, k*) is the number tag of E_{x} in Yee cell seen in Fig. S3.

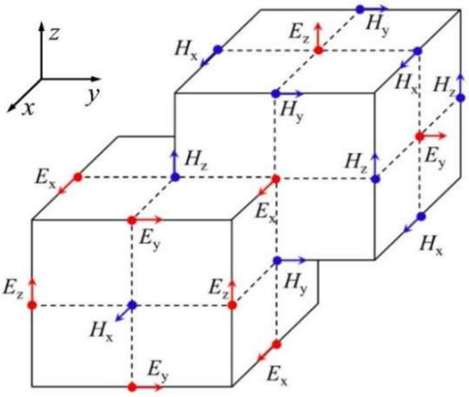

Figure S3 Schematic diagram of Yee cell grid structure.

In order to better interpret the influence of wavelength on the size effect at the micro/nano scale, the magnetic field strength and electric field direction at the z-direction cross section of different models when porosity φ =0.8 at different wavelength as: d_H = 5 μm, λ = 5 μm; d_H = 5 μm, λ = 10 μm; d_H = 5 μm, λ = 20 μm; d_H = 10 μm, λ = 5 μm; d_H = 10 μm, λ = 10 μm; d_H = 10 μm, λ = 20 μm; d_H = 20 μm, λ = 5 μm; d_H = 20 μm, λ = 10 μm; and d_H = 20 μm, λ = 20 μm are presented respectively in Fig. S4 (a) - S4 (i).

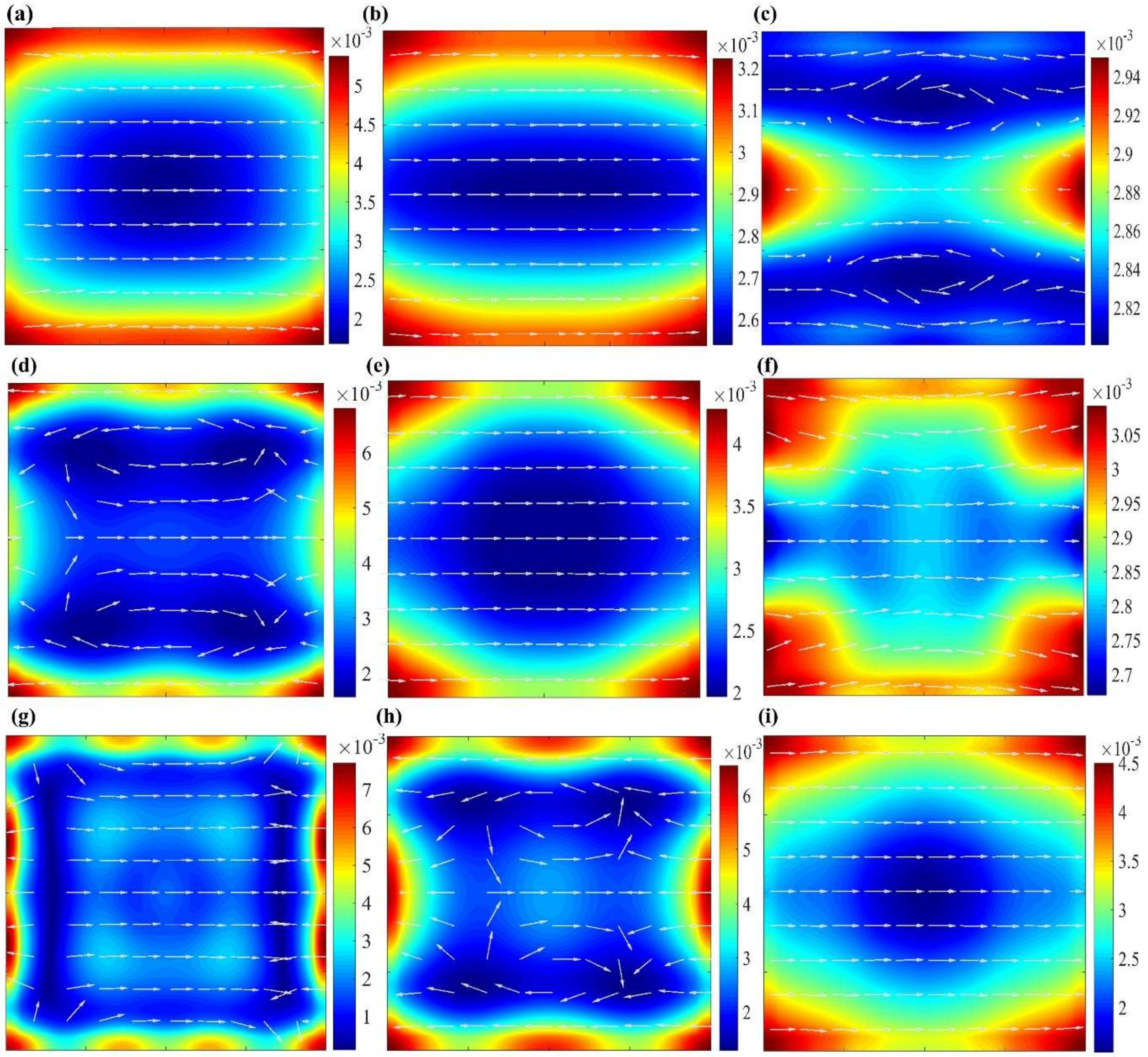

Figure S4. Magnetic field strength and electric field direction at the z-direction cross section of different models at different wavelength: (a) d_H = 5 μm, λ = 5 μm; (b) d_H = 5 μm, λ = 10 μm; (c) d_H = 5 μm, λ = 20 μm; (d) d_H = 10 μm, λ = 5 μm; (e) d_H = 10 μm, λ = 10 μm; (f) d_H = 10 μm, λ = 20 μm; (g) d_H = 20 μm, λ = 5 μm; (h) d_H = 20 μm, λ = 10 μm; and (i) d_H = 20 μm, λ = 20 μm.

As it can be seen from Fig. S4 (a), S4 (e), and S4 (i), when the cell size d_H is closed to the wavelength λ, the radiant energy dissipation is mainly distributed inside the dielectric framework, and the dissipation in the air pores is relatively low. It can be seen from Fig. S4 (b) and S4 (f), when $d_H < \lambda$, the high dissipation shifts from a uniform distribution on the four framework sides to the specific points. At the same time, the low dissipation position shifts from the pore's center to the frameworks' sides along the electric direction. In addition, when $d_H << \lambda$, weak electric vortexes are formed inside the air pore, shown in Fig.S4(c). What's more, when $d_H > \lambda$, the high dissipation shifts from a uniform distribution on the framework's sides to

multiple specific points, as seen in Fig.S4 (d) and S4(h), while the low dissipation position shifts from the pore's center to multiple low dissipation points inside the air pore. Meanwhile, electric vortexes are formed along the interface of gas and solid framework. As it can be seen in Fig. S4(g), when $d_H >> \lambda$, electric vortexes are formed inside the skeleton.

The Effect of Cellular Shapes

The influence of the cellular shape on the thermal conductivity of porous materials was illustrated by analyzing heat transfer through four porous geometries, which is shown in Fig. S5, including S4 (the cellular shape is quadrangular prism), S6 (the cellular shape is hexagonal prism), S8 (the cellular shape is octagonal prism) and SC (the cellular shape is cylinder). At the same total volume of each unit cell, the difference in the internal surface area among these four cellular structures was less than 0.62 %. For instance, when d_H = 100 nm, the internal surface areas of these four models S4, S6, S8 and SC were 51,704 nm^2, 51,710 nm^2, 51,713 nm^2 and 51,706 nm^2, respectively. The study was conducted at T = 300 K and φ = 0.80.

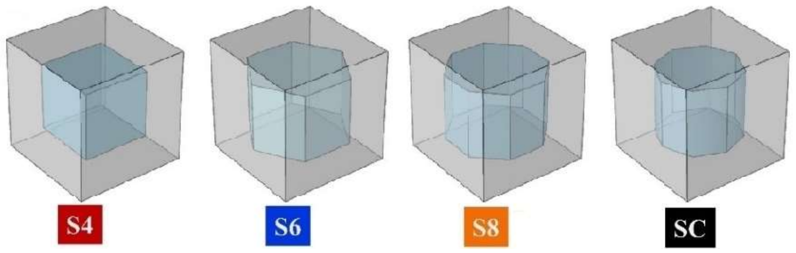

Figure S5. Schematic diagram of the different pore shape: (a)S4 model; (b)S6 model; (c)S8 model; (d) SC model.

As Fig. S6 (a) and S6(c) show, there was insignificant difference between the radiative and the total conductivities for all four models, less than 0.005 $W\cdot m^{-1}\cdot K^{-1}$, at the same radiant output power. Also, there was no significant effect of the pore shape on κ_{cond}, as seen in Fig. S6 (b). Thence, it can be concluded that the cellular shape has almost no effect on the thermal conductivity of micro/nano closed cell porous dielectric materials.

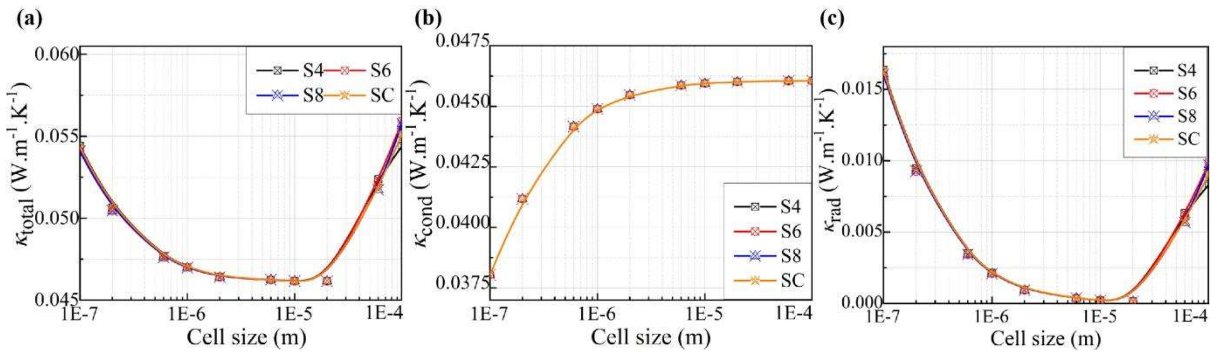

Figure S6. The thermal conductivities versus cell size at different pore shapes: (a) the total thermal conductivity; (b) the conductive thermal conductivity; (c) the radiative thermal conductivity.

The Effect of VSSA

To illustrate the effect of surface area on the thermal conductivity of the porous material with φ = 0.80, the internal surface area of the SC model was varied by adjusting the height of the teeth (60 teeth on the z-direction cross section). As seen in Fig. S7, five models of the cellular geometries, successively termed 1.0x, 1.25x, 1.5x, 1.75x and 2.0x with surface area of 1.0, 1.25, 1.5, 1.75, and 2.0 times that of the initial SC model, were studied at T = 300 K. It is worth noting that the effect of the pore shape on the thermal conductivity of the material was neglected based on the previous section's conclusion. The results are shown in Fig. S8.Meanwhile, the number of teeth, tooth height, inner radius, cell pore volume, cell pore surface area, and surface area multiplier for each model are shown in Table S2.

Table S2. Structural parameters of surface models with different volume ratios.

Model name	Teeth number	Tooth height	Inner radius	Cell pore volume	Cell pore surface area	Surface area multiplier
1.0x	0	0	$0.34d_H$	$0.314d_H^3$	$2.774d_H^2$	1.000
1.25x	3	$0.1d_H$	$0.331d_H$	$0.315d_H^3$	$3.457d_H^2$	1.246
1.25x	5	$0.1d_H$	$0.331d_H$	$0.315d_H^3$	$4.135d_H^2$	1.491
1.75x	7	$0.1d_H$	$0.331d_H$	$0.315d_H^3$	$4.865d_H^2$	1.753

2.0x	9	$0.1d_H$	$0.331d_H$	$0.315d_H^3$	$5.645d_H^2$	2.035

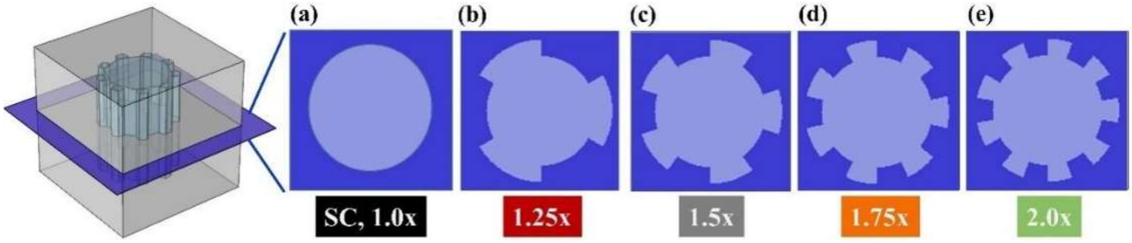

Figure S7. Schematic diagram of the (a)1.0x model; (b) 1.25x model, (c) 1.5 x model, (d) 1.75x model; and (e) 2.0x model.

As shown in Fig. S8 (a) –S8 (b), a larger surface area reduced the thermal conductivity and its conductive contributions, especially in nanocellular structures ($d_H \leq 1$ µm). Correspondingly, in Fig. S8 (a), when the surface area was doubled, the κ_{total} decreased by 0.0065 W·m^{-1}·K^{-1} at d_H = 100 nm. The decreases in conductive contributions eventually reduced κ_{total} when the surface area increased. However, as shown in Fig. S8(c), the surface area had no significant effect on κ_{rad}. In addition, the κ_{rad} of a porous closed-cell structure decreased significantly as d_H decreased. Thence, it can be concluded that the surface area has almost no effect on the thermal conductivity of micro/nano closed cell porous dielectric materials.

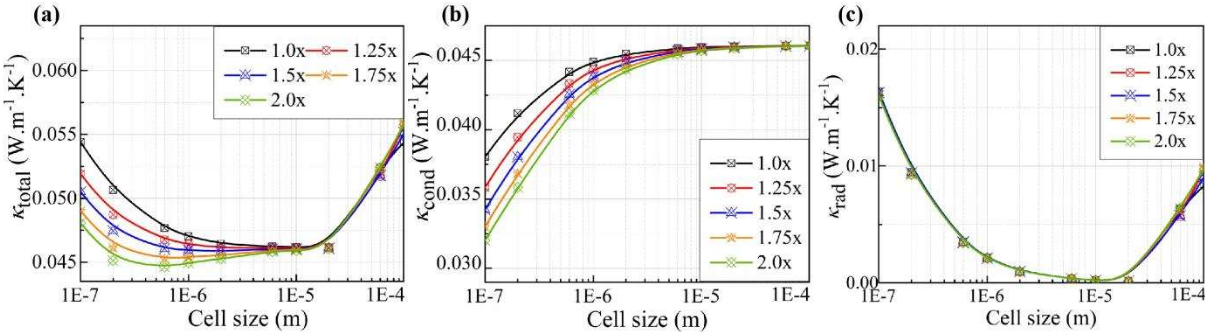

Figure S8 The thermal conductivity versus cell size at different surface areas: (a) the total thermal conductivity; (b) the conductive thermal conductivity; (c) the radiative thermal conductivity.

To sum up, the cellular shape and surface area have been treated as having no effect on the radiative thermal conductivity. In order to facilitate the calculation, in the calculation of the main text, all the pores are simplified to spheres as radiative thermal conductivity models. Therefore, the absorption coefficient and scattering coefficient can be calculated using the Mie theory, which is shown in the Eq. (15) and Eq. (16) of the main text.

S2. Settings of the Materials’ Properties

S2.1 Materials’ Refractive Index and Extinction Coefficient

The material’s refractive index and extinction coefficient of the PMMA were adopted from Tsuda’s [27] measurements, as shown in Fig. S9 (a) and S9 (b). If there is no other explanation in this paper, the refractive index and extinction coefficient of the PMMA used Fig. S9 (a) and S9 (b) for calculation.

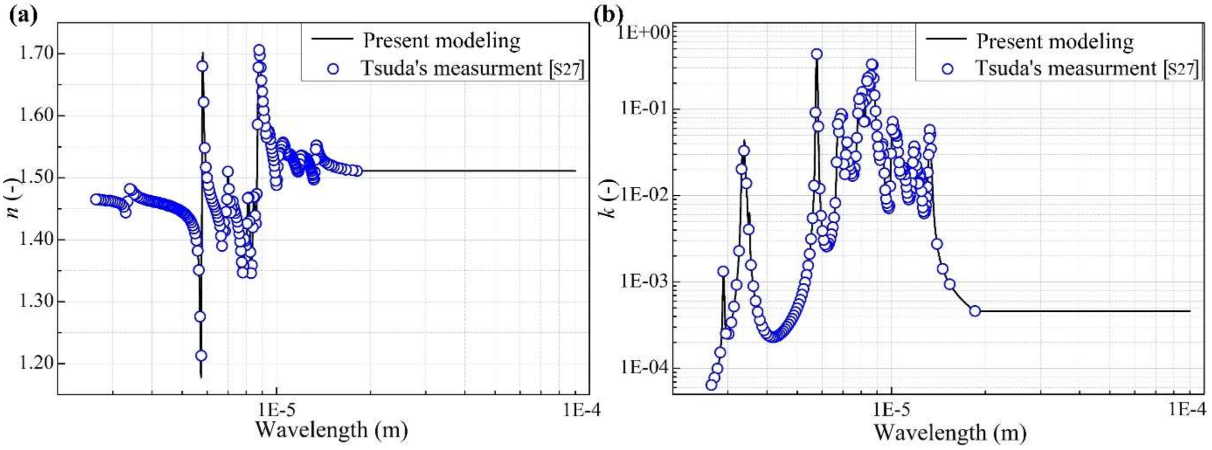

Figure S9 (a) The refractive index of PMMA; (b) the extinction coefficient of PMMA.

S2.2 Materials’ Thermal Conductivities

The thermal conductivities of pure PMMA at different temperatures adopted from Assael’s experimental measurements [S28] are shown in Fig. S10. If there is no other explanation in this paper, the thermal conductivities of the PMMA wre used as the thermal conductivity of the pure solid material κ_{bulk}. Then, the solid thermal conduction κ_{s} was calculated by a size effect modified model [S4, S29], as

$$\kappa_s = \frac{0.75\ \ w/\Lambda}{0.75\ \ w/\Lambda + 1}\kappa_{bulk} \qquad \text{(S12)}$$

where d_w is the half of the average cube structure thickness, and Λ is the phonon mean free path of the material. Since the calculated temperature range in this paper is 280K-320K, the phonon mean free path Λ of polymers [S30, S31] is much less than d_w. For ease of calculation of the polymer materials, Eq. (S12) was simplified as $\kappa_s \approx \kappa_{bulk}$. However, it is worth noting that for other dielectric materials, such as silica, Λ needed to be obtained from the handbooks and κ_s ought to calculate by Eq. (S12).

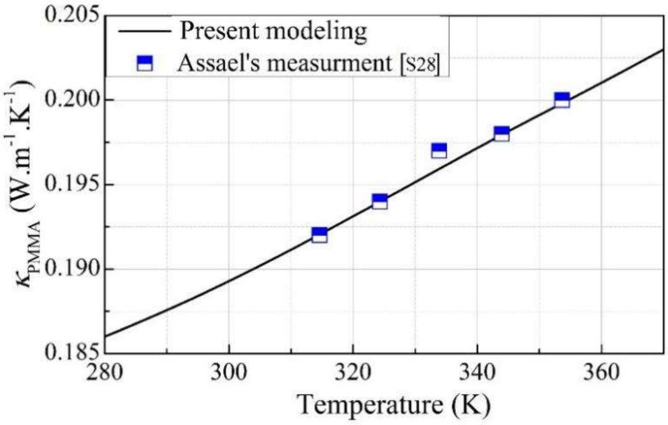

Figure S10. The thermal conductivity of PMMA with different temperature.

S3. Independence Verification

S3.1 Frequency Interval and Spatial Angular Interval

To describe the model more reasonably, the effect of the frequency interval and the spatial angular interval of the differential part of the radiant energy equation [S32, S33] on the net radiant energy density has been discussed and plotted in Fig. S11 (a) and (b).

As it can be seen from Fig. S11 (a), when the frequency interval was reduced to 2×10^{12} rad·s^{-1}, the net energy was almost the same as that at 1×10^{12} rad·s^{-1}. So, the frequency interval of 2×10^{12} rad·s^{-1} was used in the subsequent calculations. Similarly, as can be seen from Fig. S11 (b), the curves with incidence angles varied from 0º to 90º divided into 20,000 and 40,000 increments were almost coincident, so the incidence angles in this with 20,000 increments were sufficient while maintaining the accuracy.

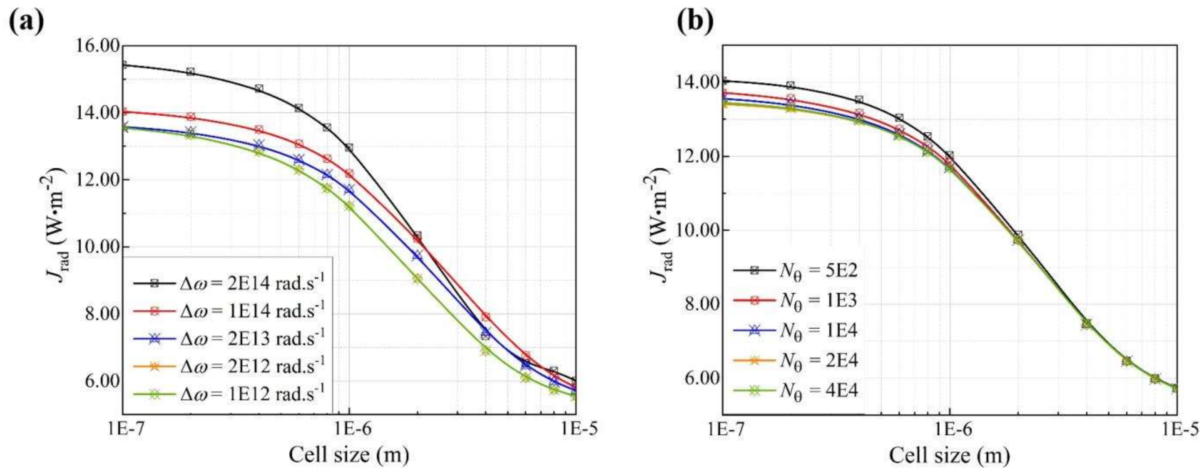

Figure S11 (a) The effect of frequency interval on net radiant energy flux density; (b) the effect of theta points on net radiant energy flux density.

S3.2 Temperature Interval

To investigate the effect of the heat transfer temperature interval on the calculation of the thermal conductivity, the effect of the temperature interval has been discussed based on non-equilibrium thermodynamics [S34, S35]. Five temperature interval models were selected as ΔT = 10K, 1K, 0.1K, 0.01K and 0.001K. The remaining parameters of five groups of models were the same, that is, the environment temperature T_L = 300 K, porosity φ = 0.80, thermal conductivity of pure solid material chosen as 0.19 W·m^{-1}·K^{-1} and the material's refractive index *n* and extinction coefficient *k* from Tsuda's [S27] experimental measurements. The results are shown in Fig. S12 (a) and (b).

For the same environment temperature, as the temperature interval increased, the radiant energy density increased corresponding to Planck's spectral distribution law [S13, S36]. Therefore, the final integrated radiant energy flux density increased, which was consistent with the trend of J_{rad} shown in Fig. S12 (a). Differentiate the net radiant energy flux to temperature, and then bring it into the calculation equation of the radiative thermal conductivity. The results show there was no significant effect of the temperature interval on the radiative thermal conductivities, as seen in Fig. S12 (b). That is, the temperature interval had little effect on the calculation of the radiative thermal conductivity. Therefore, in this research the temperature interval was selected as 1K.

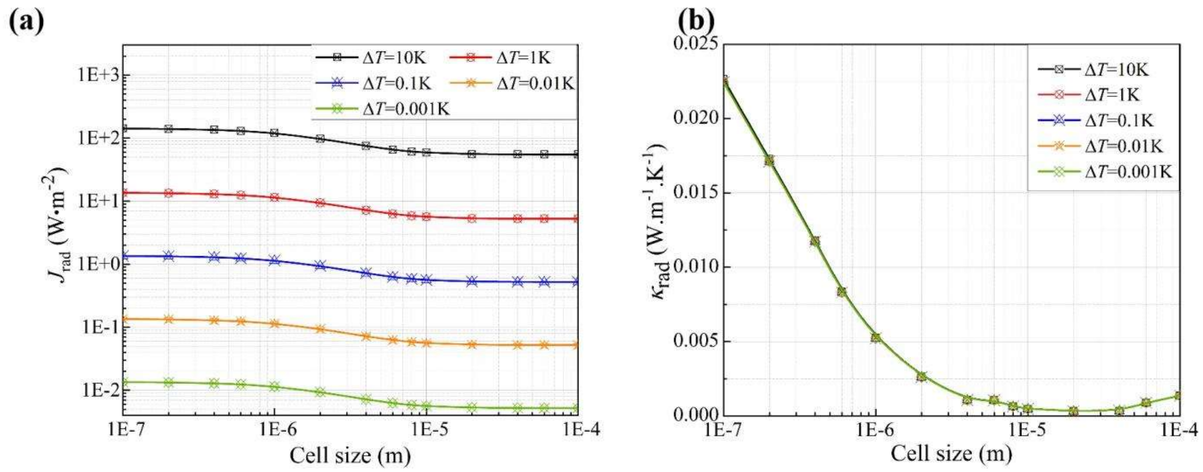

Figure S12 (a) The effect of temperature interval on net radiant energy flux density; (b) the effect of temperature interval on radiative thermal conductivity.

References

1. [S1] Shen S, Narayanaswamy A, Chen G. Surface Phonon Polaritons Mediated Energy Transfer between Nanoscale Gaps 2009. https://doi.org/10.1021/NL901208V.
2. [S2] Shen S, Henry A, Tong J, Zheng R, Chen G. Polyethylene nanofibres with very high thermal conductivities. Nat Nanotechnol 2010;5:251–5. https://doi.org/10.1038/nnano.2010.27.
3. [S3] Liu X, Wang L, Zhang ZM. Near-Field Thermal Radiation: Recent Progress and Outlook. Nanoscale Microscale Thermophys Eng 2015;19:98–126. https://doi.org/10.1080/15567265.2015.1027836.
4. [S4] Chen G. Nonlocal and Nonequilibrium Heat Conduction in the Vicinity of Nanoparticles. J Heat Transfer 1996;118:539–45. https://doi.org/10.1115/1.2822665.
5. [S5] Rytov SM, Kravt͡sov IA (I͡Uriĭ A, Tatarskiĭ VI (Valerʹi͡an I. Principles of statistical radiophysics. Springer-Verlag; 1987.
6. [S6] Zhang ZM. Nano/microscale heat transfer /. McGraw-Hill Nanosci Technol Ser 2007.
7. [S7] Yu H, Zhang H, Dai Z, Xia X. Design and Analysis of Low Emissivity Radiative Cooling Multilayer Films Based on Effective Medium Theory. ES Energy Environ 2019. https://doi.org/10.30919/esee8c333.
8. [S8] Basu S, Zhang ZM, Fu CJ. Review of near-field thermal radiation and its application to energy conversion. Int J Energy Res 2009;33:1203–32. https://doi.org/10.1002/er.1607.
9. [S9] Mulet JP, Joulain K, Carminati R, Greffet JJ. Enhanced radiative heat transfer at nanometric distances. Microscale Thermophys Eng 2002;6:209–22. https://doi.org/10.1080/10893950290053321.
10. [S10] Chapuis PO, Volz S, Henkel C, Joulain K, Greffet JJ. Effects of spatial dispersion in near-field radiative heat transfer between two parallel metallic surfaces. Phys Rev B - Condens Matter Mater Phys 2008;77:035431. https://doi.org/10.1103/PhysRevB.77.035431.
11. [S11] Sipe JE. New Green-function formalism for surface optics. J Opt Soc Am B 1987;4:481. https://doi.org/10.1364/josab.4.000481.
12. [S12] Jin S, Lim M, Lee SS, Lee BJ. Hyperbolic metamaterial-based near-field thermophotovoltaic system for hundreds of nanometer vacuum gap. Opt Express 2016;24:A635. https://doi.org/10.1364/oe.24.00a635.
13. [S13] Howell JR, Siegel R, Mengüç MP. Thermal Radiation Heat Transfer, 5th Edition. CRC Press, Taylor & Francis

Group; 2010.

14. [S14] Martín-de León J, Pura JL, Bernardo V, Rodríguez-Pérez MÁ. Transparent nanocellular PMMA: Characterization and modeling of the optical properties. Polymer (Guildf) 2019;170:16–23. https://doi.org/10.1016/j.polymer.2019.03.010.
15. [S15] Inan US, andMarshall RA, Numerical electromagnetics: the FDTD method. Cambridge University Press, 2011.
16. [S16] Guru BS, and Hiziroglu HR, Electromagnetic field theory fundamentals. Cambridge university press, 2009.
17. [S17] Zhao L, Cangellaris AC. A general approach for the development of unsplit-field time-domain implementations of perfectly matched layers for FDTD grid truncation. IEEE Microw Guid Wave Lett 1996;6:209–11. https://doi.org/10.1109/75.491508.
18. [S18] Ward AJ, Pendry JB. Refraction and geometry in Maxwell's equations. J Mod Opt 1996;43:773–93. https://doi.org/10.1080/09500349608232782.
19. [S19] Welborn SS, Detsi E. Small-angle X-ray scattering of nanoporous materials. Nanoscale Horizons 2020;5:12–24. https://doi.org/10.1039/c9nh00347a.
20. [S20] Hendrickx JMH, Borchers B, Corwin DL, Lesch SM, Hilgendorf AC, Schlue J. Inversion of Soil Conductivity Profiles from Electromagnetic Induction Measurements. Soil Sci Soc Am J 2002;66:673–85. https://doi.org/10.2136/sssaj2002.6730.
21. [S21] Li J, Liu H, Guo J, Hu Z, Wang Z, Wang B, et al. Flexible, conductive, porous, fibrillar polymer-gold nanocomposites with enhanced electromagnetic interference shielding and mechanical properties. J Mater Chem C 2017;5:1095–105. https://doi.org/10.1039/c6tc04780g.
22. [S22] Klimov V V., Pavlov AA, Guzatov D V., Zabkov I V., Savinov VD. Radiative decay of a quantum emitter placed near a metal-dielectric lamellar nanostructure: Fundamental constraints. Phys Rev A 2016;93:033831. https://doi.org/10.1103/PhysRevA.93.033831.
23. [S23] Kuppe C, Zheng X, Williams C, Murphy AWA, Collins JT, Gordeev SN, et al. Measuring optical activity in the far-field from a racemic nanomaterial: Diffraction spectroscopy from plasmonic nanogratings. Nanoscale Horizons 2019;4:1056–62. https://doi.org/10.1039/c9nh00067d.
24. [S24] Shen Y, Cheng X, Li G, Zhu Q, Chi Z, Wang J, et al. Highly sensitive and uniform surface-enhanced Raman spectroscopy from grating-integrated plasmonic nanograss. Nanoscale Horizons 2016;1:290–7. https://doi.org/10.1039/c6nh00059b.

25. [S25] Weiland T. International Journal of Numerical Modelling: Electronic Networks, Devices and Fields, 1996, 9, 295–319.
26. [S26] Mur G. Absorbing Boundary Conditions for the Finite-Difference Approximation of the Time-Domain Electromagnetic-Field Equations. IEEE Trans Electromagn Compat 1981;EMC-23:377–82. https://doi.org/10.1109/TEMC.1981.303970.
27. [S27] Tsuda S, Yamaguchi S, Kanamori Y, Yugami H. Spectral and angular shaping of infrared radiation in a polymer resonator with molecular vibrational modes. Opt Express 2018;26:6899. https://doi.org/10.1364/oe.26.006899.
28. [S28] Assael MJ, Antoniadis KD, Wu J. New measurements of the thermal conductivity of PMMA, BK7, and Pyrex 7740 up to 450K. Int J Thermophys 2008;29:1257–66. https://doi.org/10.1007/s10765-008-0504-z.
29. [S29] Obori M, Suh D, Yamasaki S, Kodama T, Saito T, Isogai A, et al. Parametric Model to Analyze the Components of the Thermal Conductivity of a Cellulose-Nanofibril Aerogel. Phys Rev Appl 2019;11:024044. https://doi.org/10.1103/PhysRevApplied.11.024044.
30. [S30] Gong P, Zhai S, Lee R, Zhao C, Buahom P, Li G, et al. Environmentally Friendly Polylactic Acid-Based Thermal Insulation Foams Blown with Supercritical CO2. Ind Eng Chem Res 2018;57:5464–71. https://doi.org/10.1021/acs.iecr.7b05023.
31. [S31] Shi W, Shuai Z, Wang D. Tuning Thermal Transport in Chain-Oriented Conducting Polymers for Enhanced Thermoelectric Efficiency: A Computational Study. Adv Funct Mater 2017;27:1702847. https://doi.org/10.1002/adfm.201702847.
32. [S32] Whitaker S. Radiant Energy Transport in Porous Media. vol. 19. 1980. Internat. Engrg. Chem. Fund. 19 (1980) 210–218.
33. [S33] Hassan MA, Abdelaziz O. Best practices and recent advances in hydronic radiant cooling systems – Part II: Simulation, control, and integration. Energy Build 2020;224:110263. https://doi.org/10.1016/j.enbuild.2020.110263.
34. [S34] Groot SR de (Sybren R, Mazur P (Peter). Non-equilibrium thermodynamics. New York : Dover Publications; 1984.
35. [S35] Winterbone DE, Turan A. Advanced Thermodynamics for Engineers: Second Edition. Elsevier Inc.; 2015. https://doi.org/10.1016/C2013-0-13437-X.
36. [S36] Gu X, Li S, Bao H. Thermal conductivity of silicon at elevated temperature: Role of four-phonon scattering

and electronic heat conduction. Int J Heat Mass Transf 2020;160:120165. https://doi.org/10.1016/j.ijheatmasstransfer.2020.120165.
